# Dirus complex species identification PCR (DiCSIP) improves the identification of *Anopheles dirus* complex from the Greater Mekong Subregion

**DOI:** 10.1186/s13071-024-06321-6

**Published:** 2024-06-16

**Authors:** Manop Saeung, Jutharat Pengon, Chatpong Pethrak, Saranya Thaiudomsup, Suthat Lhaosudto, Atiporn Saeung, Sylvie Manguin, Theeraphap Chareonviriyaphap, Natapong Jupatanakul

**Affiliations:** 1 Department of Entomology, Faculty of Agriculture, Kasetsart University, Bangkok, Thailand; 2 HSM, Univ. Montpellier, CNRS, IRD, Montpellier, France; 3 National Center for Genetic Engineering and Biotechnology (BIOTEC), Pathum Thani, Thailand; 4 Parasitology and Entomology Research Cluster (PERC), Department of Parasitology, Faculty of Medicine, Chiang Mai University, Chiang Mai, Thailand; 5 Research and Lifelong Learning Center for Urban and Environmental Entomology, Kasetsart University Institute for Advanced Studies, Kasetsart University, Bangkok, Thailand

**Keywords:** Dirus complex, Allele-specific PCR, ITS2, Misidentification, Malaria, *Anopheles*

## Abstract

**Background:**

The *Anopheles dirus* complex plays a significant role as a malaria vector in the Greater Mekong Subregion (GMS), with varying degrees of vector competence among species. Accurate identification of sibling species in this complex is essential for understanding malaria transmission dynamics and deploying effective vector control measures. However, the original molecular identification assay, Dirus allele-specific polymerase chain reaction (AS-PCR), targeting the ITS2 region, has pronounced nonspecific amplifications leading to ambiguous results and misidentification of the sibling species. This study investigates the underlying causes of these inconsistencies and develops new primers to accurately identify species within the *Anopheles dirus* complex.

**Methods:**

The AS-PCR reaction and thermal cycling conditions were modified to improve specificity for *An. dirus* member species identification. In silico analyses with Benchling and Primer-BLAST were conducted to identify problematic primers and design a new set for Dirus complex species identification PCR (DiCSIP). DiCSIP was then validated with laboratory and field samples of the *An. dirus* complex.

**Results:**

Despite several optimizations by reducing primer concentration, decreasing thermal cycling time, and increasing annealing temperature, the Dirus AS-PCR continued to produce inaccurate identifications for *Anopheles dirus*, *Anopheles scanloni*, and *Anopheles nemophilous*. Subsequently, in silico analyses pinpointed problematic primers with high Guanine-Cytosine (GC) content and multiple off-target binding sites. Through a series of in silico analyses and laboratory validation, a new set of primers for Dirus complex species identification PCR (DiCSIP) has been developed. DiCSIP primers improve specificity, operational range, and sensitivity to identify five complex member species in the GMS accurately. Validation with laboratory and field *An. dirus* complex specimens demonstrated that DiCSIP could correctly identify all samples while the original Dirus AS-PCR misidentified *An. dirus* as other species when used with different thermocyclers.

**Conclusions:**

The DiCSIP assay offers a significant improvement in *An. dirus* complex identification, addressing challenges in specificity and efficiency of the previous ITS2-based assay. This new primer set provides a valuable tool for accurate entomological surveys, supporting effective vector control strategies to reduce transmission and prevent malaria re-introducing in the GMS.

**Graphical Abstract:**

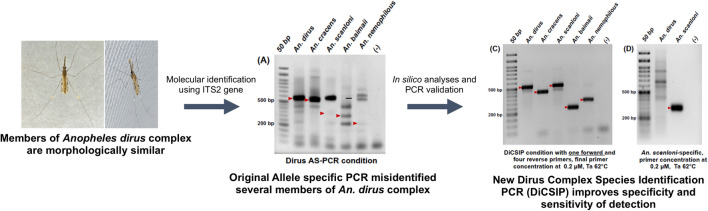

**Supplementary Information:**

The online version contains supplementary material available at 10.1186/s13071-024-06321-6.

## Background

In recent decades, countries in the Greater Mekong Subregion (GMS), particularly Thailand, have significantly reduced the number of endemic malaria cases [1]. With declining malaria cases, the focus has shifted toward malaria elimination. At this stage, entomological data become increasingly crucial to evaluate areas with potential malaria transmission and to prevent disease recurrence, especially considering the threat of zoonotic transmission of simian malaria spillover to humans [2].

Among more than 530 species of *Anopheles* mosquitoes, only ten are vectors of pathogens responsible for malaria and other vector-borne diseases [3, 4]. Certain species belong to a species complex within this genus due to the indistinguishable morphological characters of closely related sibling species. At the same time, their vectorial capacity ranges from main vectors to non-vectors. Therefore, identifying species among *Anopheles* mosquitoes to evaluate transmission risks represents a real challenge. Before advancements in molecular genetics, there were indications of misidentification at the species complex level based solely on morphological characteristics [5–8]. Subsequently, genetic information-based techniques have been employed to complement morphological identification. Polymerase chain reaction (PCR) assays has been developed and utilized for identifying mosquitoes within *Anopheles* complexes such as *Anopheles dirus*, *Anopheles minimus*, *Anopheles barbirostris*, and *Anopheles leucosphyrus* [9–12]. The internal transcribed spacer 2 (ITS2) is widely utilized as the primary DNA region. It mainly contains conserved regions suitable for designing universal primers and offers sufficient variability to distinguish even closely related species [13]. Another advantage of the ITS2 gene over other commonly used molecular markers is the ability to differentiate species on the basis of different amplicon sizes, thus eliminating the need for costly sequencing.

Members of the *Anopheles dirus* complex have been considered a medically important taxa in the GMS due to their ability to transmit *Plasmodium* parasites, pathogens responsible for human malaria [1, 14, 15]. The taxonomic hierarchy of the Dirus complex was recognized as belonging to subgenus *Cellia*, Neomyzomyia Series, Leucosphyrus Group, Leucosphyrus Subgroup [16]. The distribution range of the Dirus complex is mainly in Southeast Asia, including Thailand [17]. The complex comprises eight sibling species, six of which can be found in the GMS, including *An. dirus* (former *An. dirus* A), *Anopheles cracens* (former *An. dirus* B), *Anopheles scanloni* (former *An. dirus* C), *Anopheles baimaii* (former *An. dirus* D), *Anopheles nemophilous* (former *An. dirus* F), and *Anopheles* aff. *takasagoensis* [16]. For the other two sibling species, *An. elegans* is present in southern India, while *An. takasagoensis* has only been found in Taiwan [4, 18]. In Thailand, five species are present, except *An.* aff. *takasagoensis,* which is restricted to northern Vietnam [19]. Among all eight species, two are recognized as significant malaria vectors, namely *An. dirus* and *An. baimaii* [20–23], while the others are secondary or non-vector. Furthermore, the Dirus complex has been reported to be a vector of simian malaria, *P. knowlesi*, which is currently causing concern about zoonotic transmission in the GMS countries [1, 24, 25]. This presents a new challenge, as parasites can exist and circulate in the forest, compromising malaria elimination efforts in the region. Due to overlapping larval habitats and sympatric occurrence of the five sibling species found in Thailand, more is needed to distinguish them than relying on ecological characteristics and/or morphological characters. Therefore, molecular-based techniques are essential for the precise identification of primary malaria vectors and for categorizing risk areas on the basis of the geographical distribution of these vectors [20].

The *An. dirus* complex allele-specific multiplex PCR (Dirus AS-PCR), targeting the ITS2 gene, was designed to differentiate five sibling species of the Dirus complex in the GMS. The assay uses one universal forward primer (D-U) and four species-specific reverse primers, namely D-AC (specific to *An. dirus* and *An. scanloni*), D-B (*An. cracens*), D-D (*An. baimaii*), and D-F (*An. nemophilous*). Since its development in 1999, this technique has been adopted for entomological surveys in this region [9–12]. However, difficulties in the interpretation of PCR results due to nonspecific or inefficient amplification have been reported [26].

In this study, we describe efforts to optimize reaction conditions for the Dirus AS-PCR to improve specificity. Additionally, we performed in silico analyses, including binding site identification and amplicon predictions, to identify the causes of difficulties in the assay. Furthermore, we developed a new PCR assay called Dirus complex species identification PCR (DiCSIP) to enhance the reproducibility and sensitivity of *An. dirus* complex identification.

## Mthods

### *Anopheles dirus* complex samples

Laboratory colonies and field-derived mosquitoes belonging to the *An. dirus* complex were used in this study. *An. dirus* samples were obtained from the insectary at the Department of Entomology, Faculty of Agriculture, Kasetsart University (KU). *An. cracens* laboratory samples were obtained from the insectary of the Armed Forces Research Institute of Medical Sciences (AFRIMS) and Chiang Mai University. Field-collected *An. dirus* complex samples were obtained from previous field surveys conducted in Kanchanaburi, Prachinburi, Ranong, and Sisaket Provinces, Thailand. The samples were morphologically identified using a pictorial key under stereomicroscope [21]. A permit for field mosquito collection was obtained from the Provincial Public Health Office (permit no. 0032.006/6297). Mosquitoes were collected using human landing collection (HLC) due to the high anthropophilic behavior of these species [17]. The HLC protocol was approved by the Research Ethics Review Committee for Research Involving Human Research Participants, Kasetsart University (Certificate of Approval no. CAO63/035). Due to the need for field samples of *An. scanloni* and *An. nemophilous*, the gene constructs were synthesized on the basis of their ITS2 sequences obtained from the previous publication describing the original Dirus AS-PCR [9] using the Genscript service.

### DNA extraction

DNA extraction was conducted using either the Zymo Quick-DNA extraction kit (Zymo Research, Cat# D3024) for high-purity DNA or DNAzol-direct Reagent (MRC Inc, Cat# DN131) for high-throughput DNA extraction with field samples following the manufacturers’ protocol.

For the Zymo Quick-DNA extraction kit, the whole body of each mosquito was homogenized using 0.5 mm sterile glass beads with Bullet Blender homogenizer (NextAdvance) or plastic pestles in 500 µL of genomic lysis buffer. The debris was pelleted by centrifugation at 12,000 × *g* for 5 min, and the supernatant was transferred to a Zymo-Spin III filter in the collection tube and centrifuged at 8000 × *g* for 1 min. Afterward, 1200 µL Genomic Lysis Buffer was added to the filtrate in the collection tube. Then, the mixture was transferred to a Zymo-Spin IICR column in a collection tube and centrifuged at 10,000 × *g* for 1 min. DNA on the column was washed with 200 µL of DNA Pre-Wash Buffer, followed by 500 µL of g-DNA Wash Buffer. Finally, the column DNA was eluted with 50 µL of DNase/RNase-free water. DNA concentration was measured using the Qubit dsDNA HS Assay Kit (Invitrogen, Cat# Q32851). The eluted DNA was used immediately or stored at −20 °C until further analysis.

DNAzol-direct reagent was used to process laboratory and field samples for Dirus AS-PCR and DiCSIP assay validation. Briefly, two mosquito legs were homogenized in 30 μL of DNAzol-direct reagent using 0.5 mm sterile glass beads with Bullet Blender homogenizer (NextAdvance) or plastic pestles. Tissue debris was pelleted by centrifugation at 8000 × *g* for 3 min, and the supernatant was used directly in PCR reactions or stored at −20 °C until further use.

### PCR conditions and optimization

#### Original Dirus AS-PCR condition

The Dirus AS-PCR was conducted according to a published protocol [9]. Briefly, PCR was set up in the total reaction volume of 12.5 μL containing a 2.5 ng of genomic DNA (gDNA) or 0.1 ng of DNA from plasmid harboring ITS2 gene (pDNA), 1X Gotaq Buffer, 1.25 units of Promega GoTaq Flexi DNA Polymerase (Promega, Cat# M8295), 2 mM MgCl_2_, 10% Dimethyl sulfoxide (DMSO), 0.2 mM dNTPs, and 1 μM each of primer D-U, D-AC, D-B, D-D, and D-F. Invitrogen Taq DNA polymerase (Invitrogen, Cat# 11615-010) was also used to set up a PCR reaction by replacing the reaction buffer and enzyme while maintaining other reagents. The PCR reaction was prepared on ice to prevent nonspecific amplification during the reaction setup. The thermal cycling condition consists of 5 min denaturation step at 94 °C, followed by 35 amplification cycles of denaturation at 94 °C for 1 min, annealing temperature (Ta) at 51 °C for 1 min, extension at 72 °C for 2 min, and a final extension step at 72 °C for 10 min.

PCR products were separated and visualized using 2% agarose gel electrophoresis in 1X Tris-acetate-EDTA (TAE). A volume of 3 µL of each PCR product was mixed with 5X DNA loading dye then individually loaded into each well, and 1.5 µL of a 50 bp DNA ladder (SMOBIO, Cat# DM1100) was used as a marker. The agarose gel was run for 28 min at 100 V with a Mupid-exU electrophoresis system. Then, PCR products on agarose gel were stained with Ethidium bromide for 4 min and visualized under UV light using Syngene G:Box Chemi-XX9-F0.8 imager.

#### Modifications of the original Dirus AS-PCR

Reducing the primer concentration: the Dirus AS-PCR was conducted as described in Original Dirus AS-PCR condition, but the final primer concentration was decreased from 1 μM to 0.2 μM. Thermal cycling was also shortened with denaturation at 94 °C for 2 min, followed by 35 cycles of denaturation at 94 °C for 20 s, annealing with Ta increased from 51 °C to 55 °C or 60 °C for 20 s, and extension at 72 °C for 1 min, followed by final extension step at 72 °C for 5 min. Specific information on the reaction setup and thermal cycling conditions are described within each particular experiment.

### Bioinformatic analyses and primer design

Primer-BLAST [27] was used to analyze the potential amplicon and species specificity of the universal forward and species-specific reverse primer pairs against the nucleotide (nr) database of the *Anopheles* genus (tax id: 7164) in the National Center for Biotechnology Information (NCBI). The parameters for the alignment were set with a product size range of 70–1000 bp and melting temperatures (Tm) at 60 ± 3 °C (Table 1).

**Table 1:** Bioinformatic analyses of Dirus AS-PCR primers

Primer	Direction	Sequence (5′ → 3′)	bp	Tm (°C)	% GC	Specificity within Dirus complex	
						Expected size (product size)	Primer-BLAST results (product size)
D-U	Forward	CGCCGGGGCCGAGGTGG	17	68.22	88.24	–	–
D-AC	Reverse	CACAGCGACTCCACACG	17	58.02	64.71	*An. dirus* (562 bp) *An. scanloni* (349 or 353 bp)	*An. dirus* (353 or 345 bp) *An. baimaii* (343 bp)^a^
D-B	Reverse	CGGGATATGGGTCGGCC	17	59.26	70.59	*An. cracens* (514 bp)	*An. cracens* (514 bp)
D-D	Reverse	GCGCGGGACCGTCCGTT	17	65.62	76.47	*An. baimaii* (306 bp)	*An. baimaii* (306 bp)
D-F	Reverse	AACGGCGGTCCCCTTTG	17	59.93	64.71	*An. nemophilous* (223 bp)	No ITS2 sequence in the database

The primer properties were predicted in Benchling online tools and specificity for PCR amplification was analyzed in Primer-BLAST. The search criteria were 70–1000 bp product size, melting temperatures (Tm) at 60 ± 3 °C, Anopheles (taxid: 7164)

^a^Indicated samples of *An. dirus* misidentified as *An. baimaii*

An online tool called Benchling (https://www.benchling.com/) was used to analyze primer properties and potential binding sites and explain inconsistencies in PCR results. Furthermore, new primers were designed using a primer design tool on Benchling. The DNA sequences of *An. scanloni* and *An. nemophilous* were retrieved from published articles [9]. DNA sequences of *An. dirus* (Accession number: MW647457), *An. cracens* (Accession number: MG008574), and *An. baimaii* (Accession number: MN152993) were obtained from the GenBank database. Multiple sequence alignments (MSA) were conducted to determine regions appropriate for a new universal forward primer, as well as species-specific forward and reverse primers. The parameters for primer design include: (i) nucleotide length with 16–22 bases, (ii) G/C content of 57–77%, (iii) melting temperature (Tm) of 50–63 °C (Table 2). A new universal forward primer (DiCSIP-Uni-Fwd) was designed at the conserved region among the five species of the *An. dirus* complex. At the same time, new species-specific reverse primers (DiCSIP-Rev) were designed at distinct areas for each species. Due to the high similarity between *An. dirus* and *An. scanloni* ITS2, several forward and reverse primers were designed to differentiate these two species (Table S1). Potential binding sites of each primer on *An. dirus* complex ITS2 were analyzed on Benchling. Additionally, primer-BLAST was used to identify potential amplicons from new primers, as described above (Table 2).

**Table 2:** Sequences and bioinformatic analyses of the new DiCSIP primers to distinguish five member species within the *An. dirus* complex

Assay	Primer	Direction	Sequence (5′ → 3′)	bp	Tm (°C)	% GC	Specificity within Dirus complex	
							Expected size (product size)	Primer-BLAST results (product size)
**DiCSIP**	DiCSIP-Uni-Fwd	Forward	GAGTGATGGATACAGAGCGGG	21	56.3	57.14	–	–
	DiCSIP-Rev-AC	Reverse	ATCACTCCACCTGACCGGCAAC	22	60.88	59.09	*An. dirus* (521 bp) and *An. scanloni* (528 bp)	*An. dirus* (521 bp)
	D-B	Reverse	CGGGATATGGGTCGGCC	17	59.26	70.59	*An. cracens* (435 bp)	*An. cracens* (435 bp)
	D-D	Reverse	GCGCGGGACCGTCCGTT	17	65.62	76.47	*An. baimaii* (225 bp)	*An. baimaii* (225 bp)
	DiCSIP-Rev-F	Reverse	TCCGCAGCGCAGAGCG	16	60.27	75	*An. nemophilous* (305 bp)	No ITS2 sequence in the database
SSP	DiCSIP-Fwd-C	Forward	GCTCCCACACACACACAC	18	55.4	61.11	–	–
	DiCSIP-Rev-AC	Reverse	ATCACTCCACCTGACCGGCAAC	22	60.88	59.09	*An. scanloni* (300 bp)	No ITS2 sequence in the database

Primer properties were predicted in Benchling online tools and specificity for PCR amplification were analyzed in Primer-BLAST. The search criteria were 70–1000 bp product size, melting temperatures (Tm) at 60 ± 3 °C, *Anopheles* (taxid: 7164)

*SSP* Scanloni-specific PCR

### Validation of new DiCSIP primers

After in silico analyses, new primers were validated for specificity by PCR as follows:

#### DiCSIP universal forward primer

The DiCSIP-Uni-Fwd primer was validated with four original Dirus AS-PCR reverse primers [9] to confirm whether the replacement of the forward primer improves specificity. The DiCSIP-Uni-Fwd primer was validated by multiplex PCR with four original Dirus AS-PCR species-specific reverse primers: D-AC, D-B, D-D, and D-F. PCR reaction was performed in a volume of 12.5 μL in a final content of 2.5 ng gDNA or 0.1 ng pDNA, 1X Gotaq Buffer, 1.25 units of Promega GoTaq Flexi DNA Polymerase, 2 mM MgCl_2_, 10% Dimethyl sulfoxide (DMSO), 0.2 mM dNTPs, and 0.2 μM of each primer. The thermal cycling condition consists of 2 min denaturation step at 94 °C, followed by 35 amplification cycles of denaturation at 92 °C for 20 s, annealing temperature (Ta) 60 °C for 20 s, extension at 72 °C for 1 min, and a final extension step at 72 °C for 5 min.

#### DiCSIP species-specific reverse primers

On the basis of the data from bioinformatic analysis (Table 1) and preliminary results, it was found that reverse primers D-AC and D-F caused difficulties in species identification. After designing the new DiCSIP species-specific reverse primers, they were used in a single-plex PCR reaction together with the DiCSIP-Uni-Fwd primer using the same reaction setup and thermal cycling conditions as described in the DiCSIP universal forward primer section, except for a reduction of DMSO final concentration from 10% to 4%.

#### Scanloni-specific PCR (SSP)

*An. scanloni*-specific forward primer (DiCSIP-Fwd-C) was validated together with *An. dirus/scanloni*-specific reverse primer (DiCSIP-Rev-AC) using the PCR condition described in the DiCSIP species-specific reverse primers section, except for a reduction of DMSO from 10% to 4% and an increased Ta of 62 °C.

### Optimized conditions for DiCSIP assay

The DiCSIP assay consists of two PCR reactions. The first reaction is used to differentiate *An. dirus*/*scanloni*, *An. cracens*, *An. baimaii*, and *An. nemophilous* on the basis of their respective amplicon sizes. The PCR reactions consist of 1X Gotaq Buffer, 1.25 units of GoTaq® DNA Polymerase, 2 mM (MgCl_2_), 4% DMSO, 0.2 mM dNTPs, 0.2 μM of each of the following primers: DiCSIP-Uni-Fwd, DiCSIP-Rev-AC, D-B, D-D, and DiCSIP-Rev-F, and 2.5 ng gDNA template for *An. dirus*, *An. cracens*, and *An. baimaii*, or 0.1 ng pDNA template for *An. scanloni* and *An. nemophilous* ITS2 plasmids. Thermal cycling conditions consist of denaturation at 94 °C for 2 min, followed by 35 cycles of denaturation at 94 °C for 20 s, annealing at 62 °C for 20 s, and extension at 72 °C for 1 min followed by final extension step at 72 °C for 5 min.

The second PCR reaction is the SSP to differentiate *An. dirus* and *An. scanloni* using a pair of DiCSIP*-*Fwd-C and DiCSIP-Rev-AC primers, which was conducted as described in the Scanloni-specific PCR (SSP) section.

### Comparison of *An. dirus* complex identification by Dirus AS-PCR and DiCSIP in laboratory and field samples

The validation DiCSIP was performed using the conditions described in the Optimized conditions for DiCSIP assay section. The assay was validated using Taq DNA polymerase from two different manufacturers (Promega Gotaq Flexi DNA polymerase or Invitrogen Taq DNA polymerase) in two laboratories, the National Center for Genetic Engineering and Biotechnology (BIOTEC) and Kasetsart University (KU). The thermal cycler used at BIOTEC was the Biorad C1000 Touch Thermal Cycler, while the thermal cycler at KU was the Bioer LifePro Thermal Cycler. The identification efficiency of DiCSIP was compared with the Dirus AS-PCR, which was conducted following a condition as described in the Modifications of the original Dirus AS-PCR section with a Ta of 55 °C. Samples that resulted in amplicon sizes of approximately 520 bp in DiCSIP reaction were followed up by SSP to differentiate between *An. dirus* and *An. scanloni*.

## Results

### Dirus AS-PCR gives inconsistent PCR amplification even after condition optimization

Genomic DNA of three known species within the *An. dirus* complex (*An. dirus*, *An. cracens*, *An. baimaii*) and plasmids harboring synthetic ITS2 constructs for *An. scanloni* and *An. nemophilous* templates were used in the Dirus AS-PCR following the original protocol [9]. The expected ITS2 amplicon sizes for the target species are 562 bp for *An. dirus*, 514 bp for *An. cracens*, 349 or 353 bp for *An. scanloni*, 306 bp for *An. baimaii*, and 223 bp for *An. nemophilous* (Table 1).

**Fig. 1:**
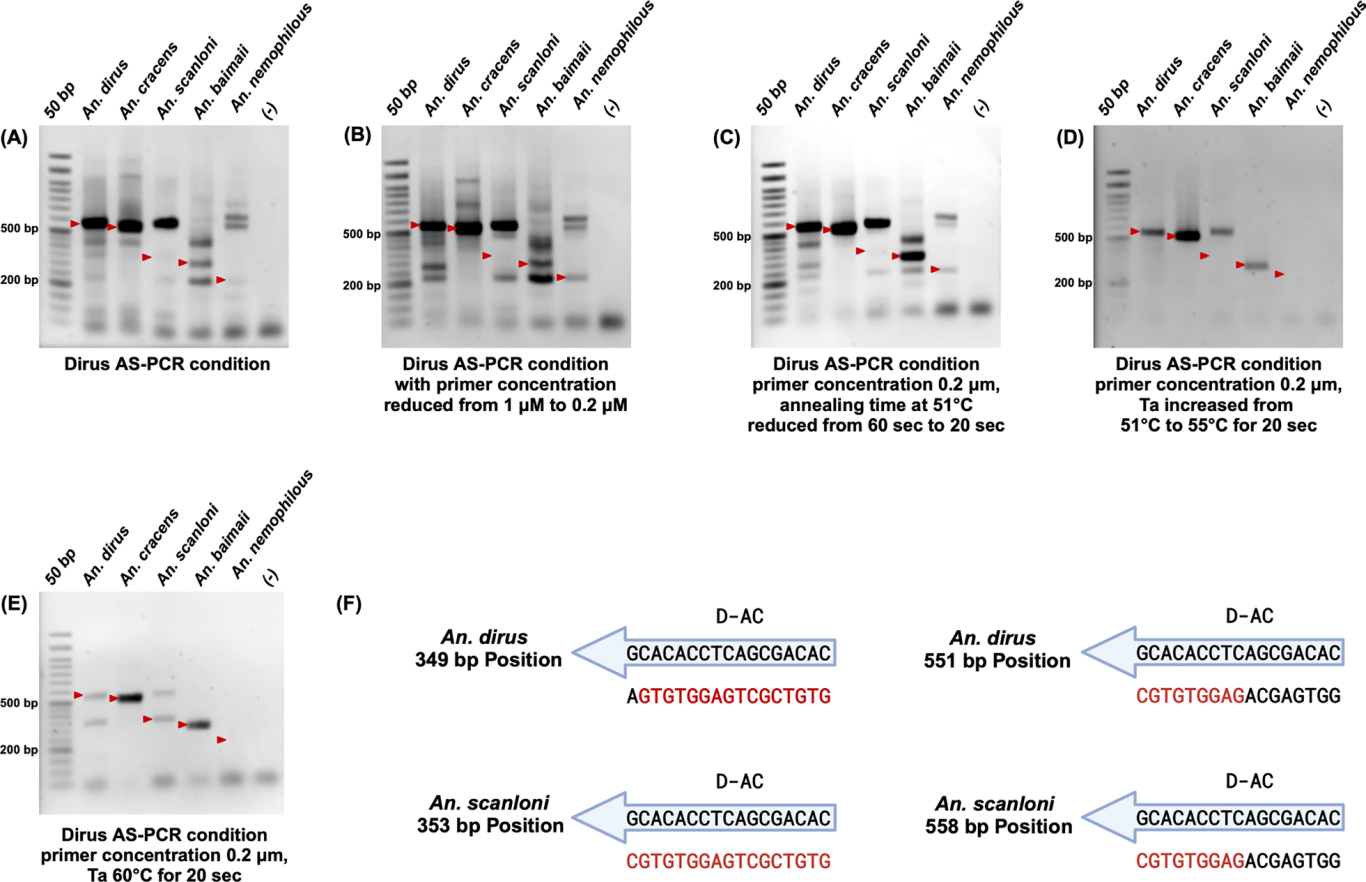
The original Dirus AS-PCR fails to correctly identify five sibling species of *An. dirus* complex present in the GMS even after optimization; agarose gel electrophoresis of the amplicons from original PCR condition (**A**), a reduction of final primer concentration (**B**), reduction of incubation time (**C**), an increase of annealing temperature to 55 °C (**D**), and an increase of annealing temperature to 60 °C (**E**). The red arrows indicate the expected sizes of PCR amplicons. **F** Diagram demonstrating D-AC binding sites on *An. dirus* and *An. scanloni* ITS2 at 349–353 bp and 551–558 bp positions. Red letters indicate matching bases between D-AC and ITS2 templates

However, following the original Dirus AS-PCR, we encountered difficulties in species identification. Despite the presence of correct amplicons in *An. dirus* and *An. cracens* samples, several nonspecific bands were present in these reactions. Moreover, we failed to obtain amplicons of the expected size for the other three species (Fig. 1A). Additionally, the *An. scanloni* sample had an amplicon of the *An. dirus* expected size, which could lead to potential misidentification of *An. scanloni* as *An. dirus*.

Given that the original Dirus AS-PCR protocol was developed more than two decades ago, variations in Taq polymerase manufacturers or thermal cyclers might cause the PCR results to differ from the original publication [9]. Therefore, we conducted Dirus AS-PCR in different laboratories (BIOTEC or KU) and utilized Taq polymerases from other manufacturers (Invitrogen or Promega). However, the results showed nonspecific and incorrect amplification (Figs. S1A and S1B).

To improve the assay’s specificity, we modified the Dirus AS-PCR conditions. As an excessive primer concentration, especially in multiplex PCR, can lead to nonspecific amplification, we first addressed this issue by reducing the final primer concentration from 1 µM to 0.2 µM, but the problems of nonspecific and incorrect amplification still persisted (Fig. 1B).

Then, we further optimized PCR condition by reducing incubation time as described in the method Modifications of the original Dirus AS-PCR section. Although shorter PCR cycling time slightly improved the specificity of *An. dirus*, *An. cracens*, and *An. baimaii*, nonspecific bands were still present under these conditions (Fig. 1C). Furthermore, we attempted to reduce nonspecific amplification by increasing the Ta from 51 °C to 55 °C, resulting in correct amplifications for *An. dirus*, *An. cracens*, and *An. baimaii* (Fig. 1D). Meanwhile, the Ta at 60 °C resulted in correct amplifications of *An. cracens* and *An. baimaii* (Fig. 1E), and we obtained two bands of expected amplicons for *An. dirus and An. scanloni at* 562 bp and 350 bp for samples of both species. These results demonstrated that the original Dirus AS-PCR primers failed to correctly differentiate between *An. dirus* and *An. scanloni*, which might lead to misidentification between these two species. In addition, although the condition in Fig. 1D demonstrated clean and correct amplicons for most species, inconsistencies were still observed with different enzyme manufacturers and thermal cyclers (Figs. S2A–S2D). We also found that the original Dirus AS-PCR primers can identify *An. dirus* and *An. scanloni* samples as *An. nemophilous* (Figs. S2A and S2B).

The results from this section confirm that the reverse primers for *An. cracens* (D-B) and *An. baimaii* (D-D) can correctly differentiate these two species after optimization of the PCR conditions, highlighting the challenges encountered with reverse primers for the other three sibling species.

### The original Dirus AS-PCR primers have multiple potential binding sites/amplicons

To identify the cause of difficulties in *An. dirus* molecular identification in the original Dirus AS-PCR, we conducted comprehensive bioinformatic analyses to identify potential off-target binding sites and nonspecific amplification for each primer using primer tools in Benchling. Additionally, Primer-BLAST was used to evaluate the off-target amplification of each primer pair, including: (i) potential off-target amplification within species, as well as (ii) off-target cross-species amplification of ITS2 gene by each pair of universal forward and species-specific reverse primer pairs against nucleotide database of *Anopheles* genus (tax id: 7164), and the results are summarized in the Table 1.

#### The D-U Dirus AS-PCR universal forward primer might cause difficulties in PCR amplification

Bioinformatic analysis of the D-U universal forward primer revealed its exceptionally high GC content at 88% and Tm at 68 °C (Table 1), with multiple potential binding sites when the PCR conditions are less stringent (Fig. S3).

#### In silico analyses reveal challenges in *An. dirus* and *An. scanloni* identification with the original D-U and D-AC primers

The in silico analyses using Benchling revealed that the D-AC primer is an exact match to *An. scanloni* ITS2, yielding a single 349 bp or 353 bp amplicon when used with D-U, while *An. dirus* ITS2 has 3′ single nucleotide mismatch at the same location (Fig. 1F, Table 1, Fig. S4). In addition to this location, the D-AC has 9 bp out of 17 bp complementary, leading to a 562 bp amplicon in both *An. dirus* and *An. scanloni* ITS2. Such a design of D-AC allows the reverse primer to differentiate between *An. dirus* and *An. scanloni* because of the presence 3′ single nucleotide mismatch in *An. dirus* lowers the amplification efficiency [28], increasing the likelihood of 562 bp amplification in *An. dirus,* while *An. scanloni* yields 349 bp or 353 bp amplicon. The in silico analysis of D-AC explains the two bands observed in the PCR reactions since both amplicons are possible for *An. dirus* and *An. scanloni*.

Primer-BLAST of the universal forward (D-U) and reverse primer (D-AC) did not provide any result for *An. scanloni* due to the unavailability of its ITS2 sequence in the NCBI database. Instead, the search returned several matches of 353 bp or 345 bp for *An. dirus* ITS2 (Fig. S4A), but not 562 bp due to insufficient partial match (only 9 bp out of 17 bp) on the latter location. Interestingly, while most *An. dirus* ITS2 sequences obtained by Primer-BLAST had this 3′ single nucleotide mismatch as expected, one of the *An. dirus* hit (OQ091691) had a complete match. This suggests a potential misidentification of *An. scanloni* as *An. dirus* in the NCBI database. The follow-up analysis by MSA between OQ091691, *An. dirus* and *An. scanloni* ITS2 from Walton et al. [9], and another *An. dirus* ITS2 from NCBI (MW647457), confirms that the OQ091691 should indeed be *An. scanloni* since the sequence lacks the deletion of CA repeats observed in *An. dirus* ITS2 (Fig. S4B).

#### In silico analyses confirms high specificity of Dirus AS-PCR primers for *An. cracens* (D-U and D-B pair) and *An. baimaii* (D-U and D-D pair) identification

In silico binding site identification revealed that the *An. cracens* specific D-B primer has potential off-target binding sites albeit with much lower Tm (45.9 °C) than the on-target binding site (56.2 °C). The *An. baimaii* specific D-D primer had no potential off-target amplification (Fig. S5). Primer-BLAST of these two species only returned on-target ITS2 gene (Table 1).

#### The off-target analysis of the original Dirus AS-PCR primers for *An. nemophilous* identification (D-U and D-F pair)

This set of primers also does not have cross or on-species off-target binding sites (Fig. S5). Primer-BLAST has yet to receive any results from *An. nemophilous* because its ITS2 sequence is not available in the NCBI database (Table 1).

### Development of new primers for Dirus complex species identification PCR (DiCSIP)

The combination of experimental and in silico analyses above provides strong evidence that universal forward (D-U) and reverse primers (D-AC and D-F) were inefficient in identifying *An. dirus*, *An. scanloni*, and *An. nemophilous*. Therefore, we designed a new set of primers for the identification of these three species and used them in a multiplex PCR assay, hereinafter referred to as the Dirus complex species identification PCR (DiCSIP).

#### Design and validation of a new universal forward primer (DiCSIP-Uni-Fwd)

**Fig. 2:**
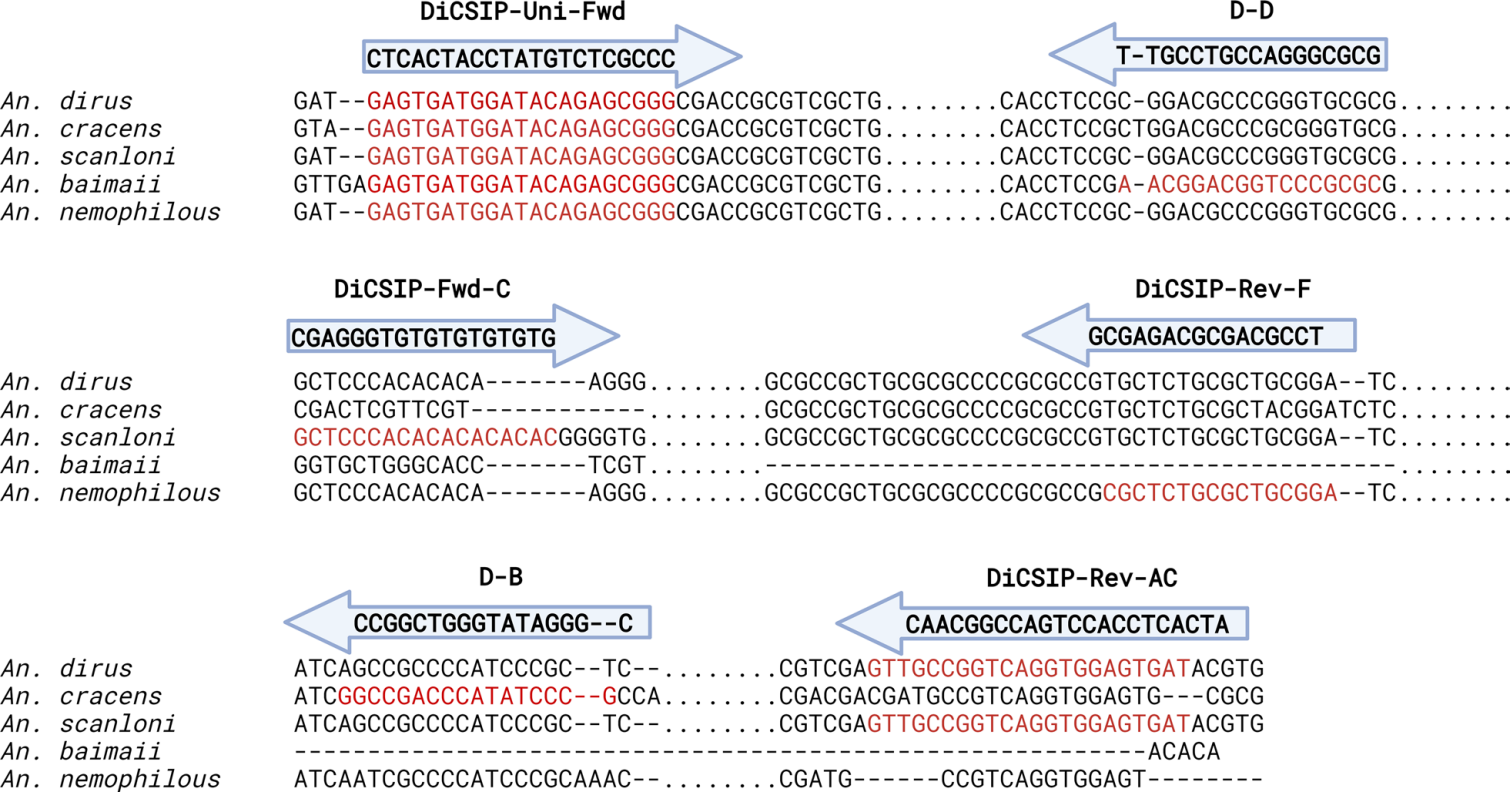
Multiple sequence alignment of ITS2 sequences of five *An. dirus* complex sibling species and binding sites of DiCSIP primers. The sequences of *An. dirus* (Accession: MW647457.1), *An. cracens* (Accession: MG008574.1), and *An. baimaii* (Accession: MN152993.1) were retrieved from NCBI, while those of *An. scanloni* and *An. nemophilous* were obtained from a published article [9]. The binding sites of each primer were highlighted in red. Dots (.) indicate shortened sequences, while dashes (–) indicate base insertion/deletion. The full alignment can be found in Fig. S6

To design the DiCSIP-Uni-Fwd primer, MSA was conducted using ITS2 sequences from five species within the *An. dirus* complex (Fig. 2, Fig. S6), and the DiCSIP-Uni-Fwd primer was designed within the conserved region of the species. The properties of the new forward primer were significantly improved (57.14% GC and 56.3 °C Tm) compared with the original D-U primer (88.24% GC and 68.22 °C Tm) (Table 2). In silico potential binding site identification revealed only one binding site on ITS2 in all five *An. dirus* complex species (Fig. S7), suggesting improved specificity compared with the previous primer (D-U).

**Fig. 3:**
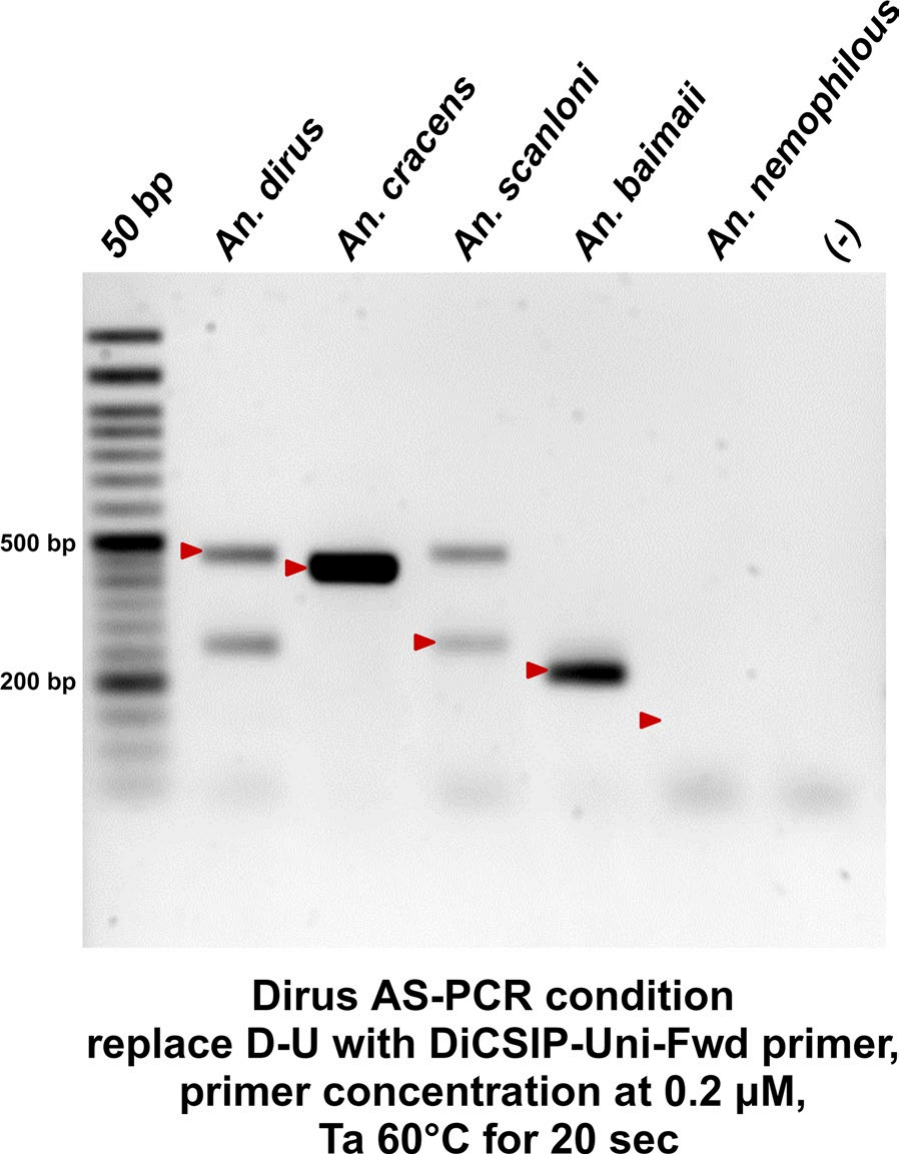
Replacement of the universal forward primer alone does not solve the problem of Dirus AS-PCR. PCR was conducted using the DiCSIP-Uni-Fwd primer and all original reverse primers (DiCSIP-Uni-Fwd, D-AC, D-B, D-D, and D-F) at final primer concentration of 0.2 μM each. The red arrow mark indicates the expected sizes of PCR amplicons

Following the in silico design of DiCSIP-Uni-Fwd, we conducted a PCR to assess whether this primer could enhance the specificity of the Dirus AS-PCR. In this experiment, the original universal forward primer D-U was replaced by the new forward primer DiCSIP-Uni-Fwd while keeping all the original reverse primers. The PCR was conducted with a final primer concentration of 0.2 μM each (DiCSIP-Uni-Fwd, D-AC, D-B, D-D, and D-F), and the same thermal cycling condition as Fig. 1E. The DiCSIP-Uni-Fwd was 79 bp downstream from the original forward primer. Thus, the expected amplicons were 483–489 bp for *An. dirus*, 435 bp for *An. cracens*, 287–294 bp for *An. scanloni*, 227 bp for *An. baimaii*, and 144 bp for *An. nemophilous*. Replacing only the forward primer could not solve the problem with *An. dirus*, *An. scanloni*, and *An. nemophilous* samples (Fig. 3). Therefore, a new set of reverse primers was needed to improve PCR species identification.

#### Design and validation of new DiCSIP primers for *An. dirus* and *An. scanloni* identification

Due to the high sequence similarity between *An. dirus* and *An. scanloni* ITS2, designing a new reverse primer that targets only *An. dirus* or *An. scanloni* ITS2 at different binding sites was impossible. Therefore, we created two sets of reverse primers, one targeting both species (DiCSIP-Rev-AC) and another targeting only *An. scanloni* (DiCSIP-Rev-C2 to C6). With these two primers in a multiplex reaction, it was expected that *An. dirus* will have one amplicon from DiCSIP-Rev-AC primer, while *An. scanloni* will have additional amplicon from DiCSIP-Rev-AC and DiCSIP-Rev-C primers.

**Fig. 4:**
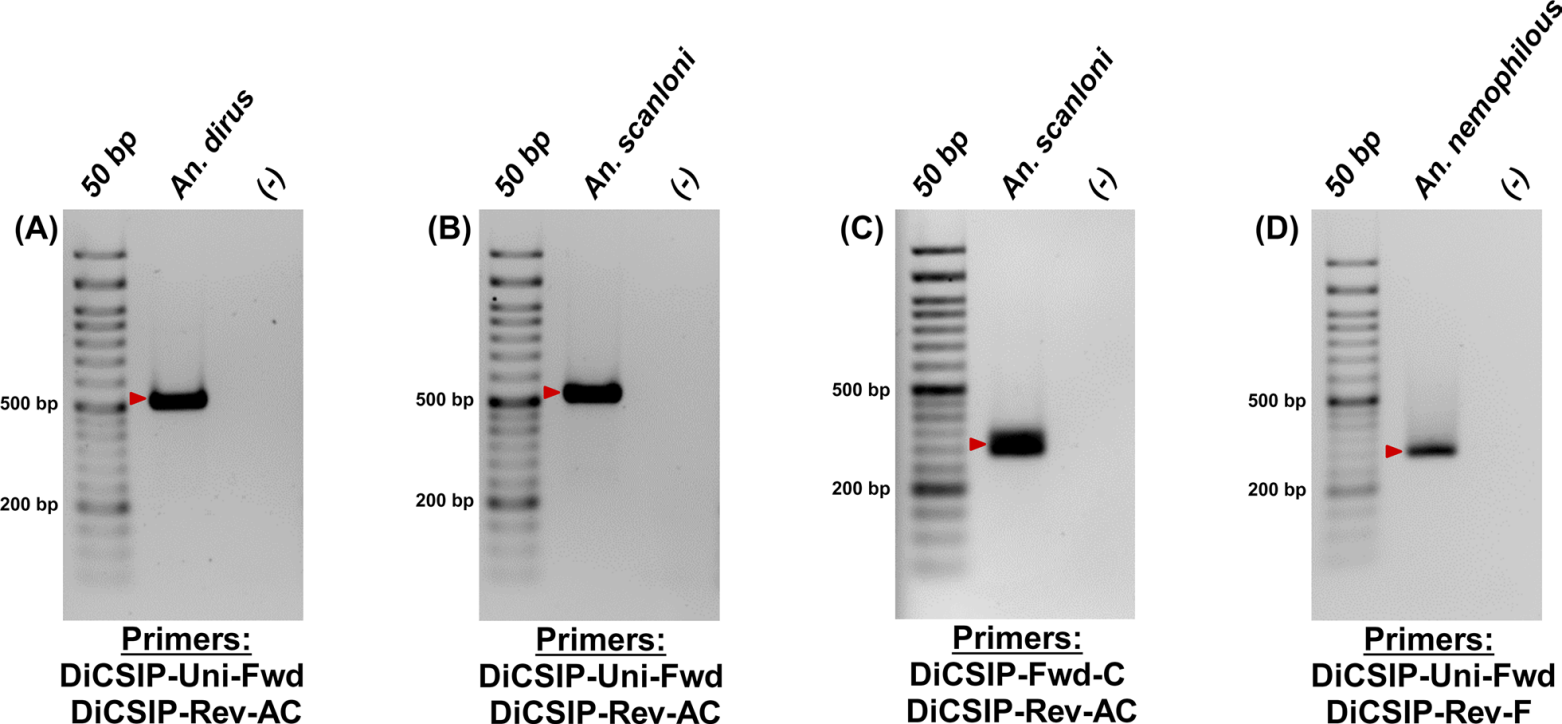
Single-plex PCR demonstrates high specificity of the new primers for *An. dirus*, *An. scanloni*, and *An. nemophilous* identifications*.* Validation of the new *An. dirus/scanloni*-specific reverse primers to identify *An. dirus* (**A**) and *An. scanloni* (**B**). Validation of the new *An. scanloni*-specific forward primer to identify *An. scanloni* (**C**). Validation of the new *An. nemophilous*-specific reverse primer to identify *An. nemophilous* (**D**). The red arrows indicate the expected sizes of PCR amplicon for each species

The DiCSIP-Rev-AC primer has a GC content of 59.09% and a Tm of 60.88 °C. The amplicon size from DiCSIP-Uni-Fwd and DiCSIP-Rev-AC primers was expected to be 521 bp for *An. dirus* and 528 bp for *An. scanloni* (Table 2). The analysis revealed high specificity with no off-target binding sites (Fig. S8). The validation by single-plex PCR containing the DiCSIP-Uni-Fwd and DiCSIP-Rev-AC primers resulted in specific amplicons of approximately 520 bp for both *An. dirus* and *An. scanloni* (Fig. 4A, B).

Six reverse primers were designed to target the *An. scanloni* ITS2 and differentiate this species from *An. dirus* with primer properties range from 57.14% GC to 68.75% GC and 55.3–61.0 °C Tm (Table S1). In silico analyses of amplicons between DiCSIP-Uni-Fwd and the six new *An. scanloni* reverse primers predicted amplicon sizes ranging from 246 bp to 272 bp (Table S1, Fig. S9). However, PCR validation demonstrated that none of the new *An. scanloni* reverse primers yielded correct amplicons (Fig. S9).

As no alternative region was available for designing *An. scanloni* ITS2-specific reverse primers, we then changed the strategy for *An. scanloni*-specific forward primer (DiCSIP-Fwd-C) (Fig. S10). Validation by PCR with the DiCSIP-Fwd-C and DiCSIP-Rev-AC primers using the *An. scanloni* ITS2 plasmid as a template showed a clean amplification at 300 bp (Fig. 4C).

#### Design and validation of new DiCSIP primer for *An. nemophilous* identification

A new reverse primer specifically targeting *An. nemophilous* ITS2 (DiCSIP-Rev-F) was designed with primer properties of 75% GC and 60.27 °C Tm. This primer has an exact match binding site to *An. nemophilous* ITS2 (Fig. S11), resulting in an expected amplicon size of 223 bp (Table 2). However, the in silico analysis suggested potential off-target binding sites of DiCSIP-Rev-F primer on *An. nemophilous* ITS2; this is the only region to design *An. nemophilous*-specific primer (Fig. S11). Nevertheless, single-plex PCR validation with DiCSIP-Uni-Fwd and DiCSIP-Rev-F primers with *An. nemophilous* plasmid exhibited a single 223 bp amplicon (Fig. 4D).

### The new multiplex DiCSIP improves the identification of *An. dirus* complex sibling species

**Fig. 5:**
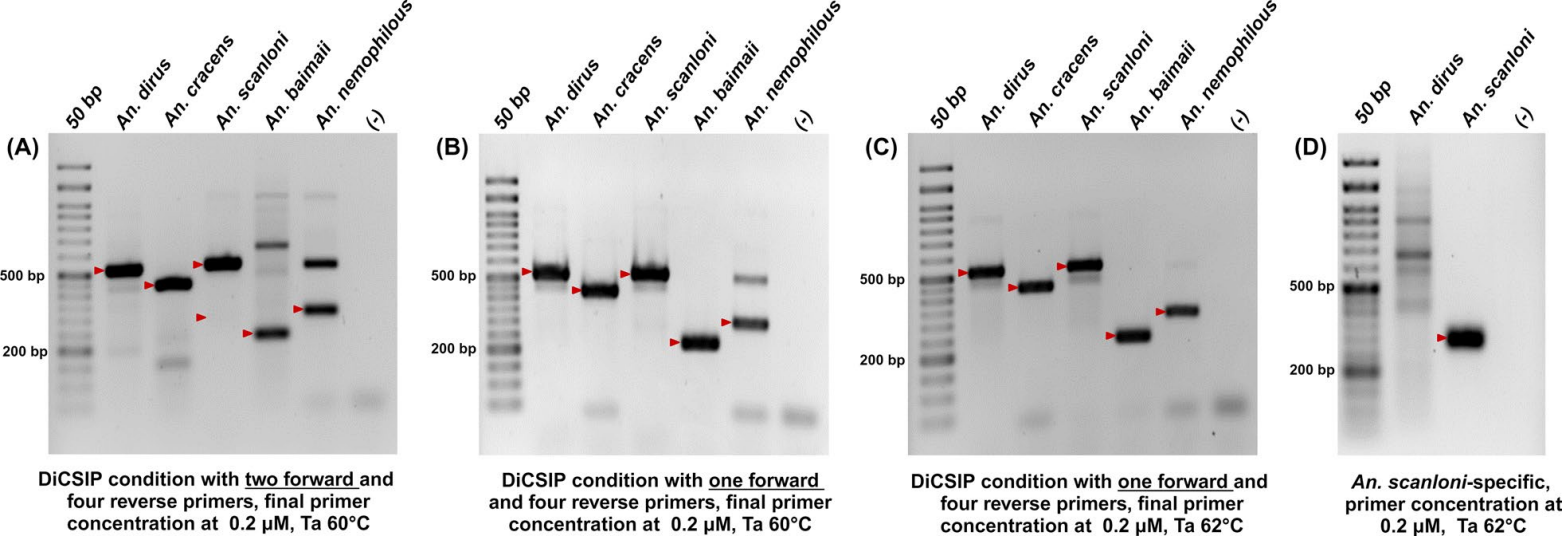
DiCSIP correctly identifies five sibling species of the *An. dirus* complex. **A** DiCSIP reaction containing two forward (DiCSIP-Uni-Fwd and DiCSIP-Fwd-C) and four reverse primers (DiCSIP-Rev-AC, D-B, D-D, and DiCSIP-Rev-F). **B** DiCSIP reaction containing one forward and four reverse primers: DiCSIP-Uni-Fwd, DiCSIP-Rev-AC, D-B, D-D, and DiCSIP-Rev-F. **C** DiCSIP reaction increasing annealing temperature from 60 °C to 62 °C using one forward and four reverse primers: DiCSIP-Uni-Fwd, DiCSIP-Rev-AC, D-B, D-D, and DiCSIP-Rev-F. **D** SSP reaction containing DiCSIP-Fwd-C and DiCSIP-Rev-AC primers correctly differentiate *An. scanloni* from *An. dirus*. The red arrow mark indicates the expected sizes of PCR amplicons

After successfully validating the specificity of the new primers in single-plex PCR, we combined all six primers, including two forward primers (DiCSIP-Uni-Fwd and DiCSIP-Fwd-C) and four reverse primers (DiCSIP-Rev-AC, D-B, D-D, and DiCSIP-Rev-F) in a multiplex PCR at a final concentration of 0.2 μM each. The amplification was conducted as described in the DiCSIP universal forward primer section. The amplicon sizes for each species are listed in Table 2. Unexpectedly, the multiplex PCR with two forward and four reverse primers in one reaction (Fig. 5A**)** could correctly identify only *An. dirus* (521 bp) and *An. cracens* (435 bp). In contrast, two amplicons were expected from *An. scanloni* sample at 521 bp and 300 bp, the actual PCR amplification only resulted in a single band at 521 bp, suggesting that the multiplex reaction might interfere with the binding of *An. scanloni*-specific DiCSIP-Fwd-C primer. In addition to the on-target amplification for *An. baimaii* (225 bp) and *An. nemophilous* (305 bp), these samples also exhibited a fainter off-target band at approximately 560 bp and 520 bp for these two species, respectively. These results suggested that DiCSIP-Fwd-C primer might interfere with other primers, making it challenging to accurately differentiate sibling species within the *An. dirus* complex using one multiplex PCR reaction. Therefore, separate PCR reactions are needed to ensure precise species identification, the first reaction with the DiCSIP-Uni-Fwd forward primer and four reverse primers (DiCSIP-Rev-AC, D-B, D-D, and DiCSIP-Rev-F) to identify *An. dirus*/*scanloni*, *An. cracens*, *An. baimaii*, and *An. nemophilous* on the basis of their respective amplicon sizes. The second Scanloni-specific PCR (SSP) assay aims to differentiate *An. dirus* and *An. scanloni* by using a pair of DiCSIP-Fwd-C and DiCSIP-Rev-AC primers with expected size at 300 bp.

The DiCSIP reaction was initially validated using a Ta of 60 °C, which accurately identify all five species, albeit with a fainter off-target band at 520 bp for *An. nemophilous* (Fig. 5B). The nonspecific band in *An. nemophilous* sample could be eliminated by increasing the Ta to 62 °C (Fig. 5C). The differentiation between *An. dirus* and *An. scanloni* samples by SSP was validated with a Ta of 62 °C, resulting in a correct amplicon at 300 bp for the *An. scanloni* sample (Fig. 5D). Although off-target amplification appeared as a smear larger than 300 bp for the *An. dirus* sample, the specificity could not be further improved, as this is the only region available for designing an *An. scanloni*-specific primer.

### The DiCSIP improves sensitivity of *An. dirus* species complex identification

**Fig. 6:**
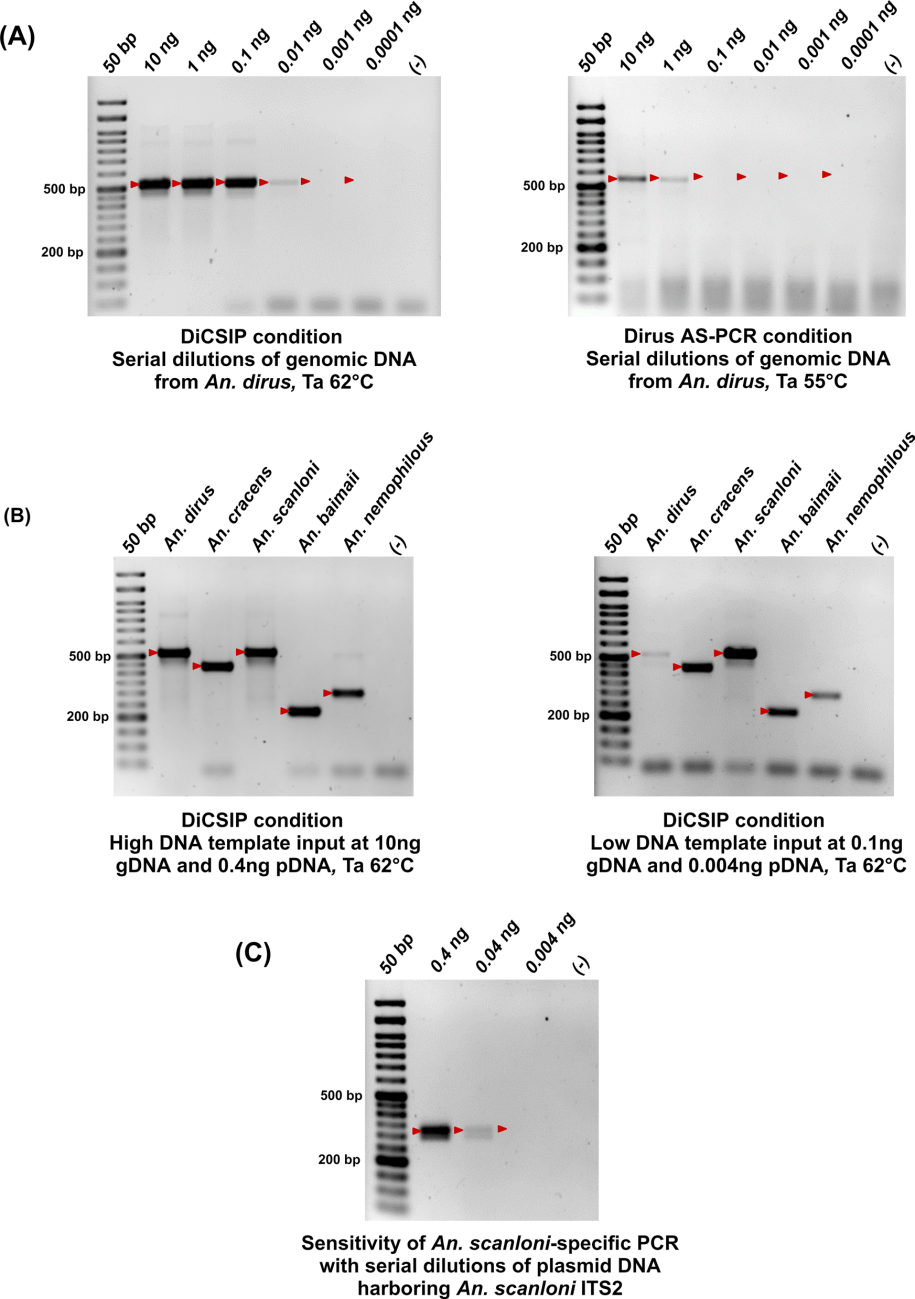
DiCSIP has higher sensitivity and a wider operational range than Dirus AS-PCR. **A** Sensitivity of DiCSIP and Dirus AS-PCR was determined using *An. dirus* gDNA ranging from 0.0001 ng to 10 ng per reaction. **B** Identification of five *An. dirus* complex sibling species with high (10 ng gDNA or 0.4 ng pDNA) and low (0.1 ng gDNA or 0.004 ng pDNA) quantity of template by DiCSIP. **C** Operational range of SSP for *An. scanloni* identification with 0.004–0.4 ng of *An. scanloni* ITS2 plasmid DNA. The red arrows indicate the expected sizes of PCR amplicons

Primers with a wide operational range and high sensitivity are crucial for reproducibility, especially across laboratories. Since *An. dirus* was the most problematic sample in the original PCR, we compared an operational range of the Dirus AS-PCR and DiCSIP by using varying concentrations of *An. dirus* gDNA template, ranging from 10 ng to 0.0001 ng/12.5 µL reaction. The results showed that the DiCSIP consistently and accurately produced the correct amplicon across a wide range of template concentrations from 10 ng to 0.01 ng (Fig. 6A). In contrast, the original Dirus AS-PCR had a much more limited working range for DNA templates, spanning from 10 ng to 1 ng (Fig. 6A). Additionally, the intensity of the amplicon from the reaction with a 10 ng template using Dirus AS-PCR was fainter than that of the 0.1 ng template using DiCSIP. These results demonstrated at least a 100-fold improvement in sensitivity by DiCSIP.

To expand the observation to other sibling species of the *An. dirus* complex, gDNA templates at 10 ng and 0.1 ng or pDNA templates at 0.4 ng and 0.004 ng were used in DiCSIP to evaluate the operational range for species identification. The results in Fig. 6B confirmed that DiCSIP effectively identifies five sibling species of the *An. dirus* complex both at high and low template quantities per 12.5 µL reaction. Similarly, the operational range of the SSP was assessed with a pDNA template, ranging from 0.4 ng to 0.004 ng/12.5 µL reaction. The results demonstrated robust amplification at 0.4 ng, while the amplicon at 0.04 ng had low intensity (Fig. 6C).

### DiCSIP outperforms Dirus AS-PCR in *An. dirus* complex identification

To demonstrate the efficiency of DiCSIP, we compared species identification between DiCSIP and the original Dirus AS-PCR on laboratory and field specimens that were morphologically identified as *An. dirus*. These tests were carried out at two different laboratories, BIOTEC and KU.

The DiCSIP consistently and correctly identified all laboratory specimens of *An. dirus* and *An. cracens* in both laboratories. However, the original Dirus AS-PCR yielded contradictory results between laboratories for *An. dirus*, specifically, the original Dirus AS-PCR correctly identified *An. dirus* specimens at KU, but the same samples were incorrectly identified as *An. nemophilous* at BIOTEC (Table 3). In contrast, all *An. cracens* laboratory specimens were correctly identified by assays in both laboratories (Table 3).

**Table 3:** Summary of *An. dirus* complex species identification by DiCSIP and Dirus AS-PCR in laboratory and field-collected specimens

Laboratory	Insectary/locality	*An. dirus*		*An. cracens*		*An. baimaii*		*An. nemophilous*	
		Original Dirus AS-PCR	DiCSIP	Original Dirus AS-PCR	DiCSIP	Original Dirus AS-PCR	DiCSIP	Original Dirus AS-PCR	DiCSIP
KU	*An. dirus* laboratory colony	10	10	–	–	–	–	–	–
	*An. cracens* laboratory colony	–	–	10	10	–	–	–	–
	Kanchanaburi Province	4	4	–	–	6	6	–	–
	Prachinburi Province	9	9	–	–	1	1	–	–
	Ranong Province	–	–	–	–	10	10	–	–
	Sisaket Province	10	10	–	–	–	–	–	–
BIOTEC	*An. dirus* laboratory colony	–	10	–	–	–	–	10^a^	–
	*An. cracens* laboratory colony	–	–	10	10	–	–	–	–
	Kanchanaburi Province	–	4	–	–	6	6	4^a^	–
	Prachinburi Province	–	9	–	–	1	1	9^a^	–
	Ranong Province	–	–	–	–	10	10	–	–
	Sisaket Province	–	10	–	–	–	–	10^a^	–

^a^The samples were identified as *An. dirus* by the DiCSIP

Next, the validation of the primers was extended to field-collected specimens morphologically identified as *An. dirus* complex, with ten specimens from each of four collection sites in Thailand: Kanchanaburi, Prachinburi, Ranong, and Sisaket Provinces. We found that the DiCSIP and the original Dirus AS-PCR provided the same identification results for all *An. cracens* and *An. baimaii* samples regardless of collection sites and laboratory conducting the identification (Table 3). However, the DiCSIP correctly identifies field-derived *An. dirus* irrespective of the laboratory performed the assay. The original Dirus AS-PCR correctly identified *An. dirus* specimens at KU, but the same samples were incorrectly identified as *An. nemophilous* at BIOTEC (Table 3).

## Discussion

Members of *An. dirus* complex present in the GMS, including Thailand, have been incriminated as vectors for human malaria, with two species, *An. dirus* and *An. baimaii*, recognized as the main malaria vectors [20–23, 29], while the three other species are identified as either secondary/incidental vectors (*An. cracens*, *An. scanloni*) or even non-vector (*An. nemophilous*) [18]. Six of the eight sibling species of the *An. dirus* complex share overlapping spatial distributions (sympatry) but exhibit distinct behaviors and vector competence. Given these complexities, precisely identifying these sibling species is very important to comprehend and accurately define local malaria transmission dynamics fully. This is critical for implementing appropriate vector control strategies and maximizing cost-effectiveness and resource utilization, mainly as Thailand aims to eliminate malaria by 2024 and the GMS aims to eliminate by 2030 [30]. Even after elimination, accurate vector surveillance is essential to monitor the potential reintroduction of malaria to the GMS. Accurate surveillance is also crucial in combating the emerging problem of zoonotic transmission of simian malaria. An accurate multiplex PCR method that can differentiate *An. dirus* complex species on the basis of amplicon sizes provide a cheap entomological surveillance tool for vector ecology and malaria epidemiology. However, the previously described Dirus AS-PCR protocol may provide inconsistent results and cause misidentification without proper PCR control.

Primer-BLAST results using previously described Dirus AS-PCR primers raised our concern for previous misidentification of sibling species of the *An. dirus* complex. We found that some of the *An. dirus* ITS2 in the NCBI was misidentified, and should actually be *An. scanloni*. Without *An. scanloni* ITS2 available in the database, the future BLAST search of *An. scanloni* ITS2 sequence will return that *An. dirus* accession and might lead to false identification (Fig. S4**)**. Without careful analysis, subsequent study of the misidentified samples might lead to false advancements in research on *An. dirus* complex, as well as incorrect information on the geographical distribution of each species. In addition to misidentification between *An. dirus* and *An. scanloni*, we also found a misidentification of *An. dirus* as *An. baimaii* in several NCBI entries (Fig. S12B). Therefore, a comprehensive revision of *An. dirus* complex ITS2 in NCBI is needed to improve the accuracy of future species identification.

The original molecular identification assay, Dirus AS-PCR, was developed more than two decades ago to differentiate five sibling species of the *An. dirus* complex found in the GMS [9]. Since the development of the Dirus AS-PCR for *An. dirus* complex, several articles demonstrated adaptations of this protocol to identify the sibling species [26, 31–40]. Despite the need for accurate entomological information, we found that the existing Dirus AS-PCR [9] yielded inconsistent results of *An. dirus*, *An. scanloni*, and *An. nemophilous* in our laboratory compared with the original article. In addition to the results demonstrated in our study, inaccurate identification was also described by Monthatong [26], who demonstrated challenges in correctly identifying *An. dirus* and *An. scanloni*. Since the ITS2 region has secondary structures and complementary sequences throughout the gene, the development of PCR diagnostic based on ITS2 can be complex, and slight changes in reaction components, conditions, and equipment may affect identification accuracy. Indeed, our study demonstrated that the difference in thermal cycler used for PCR can cause misidentification.

In addition to the differences in technical aspects, we should also consider a biological aspect, such as the sequences used for primer design in the previous study, which might not adequately represent the full range of genetic diversity within these species or the ITS2 of the *An. dirus* complex may have evolved. Indeed, we found that the *An. dirus* ITS2 sequence described in Walton et al. (1999) was slightly different from the more recent *An. dirus* ITS2 (Fig. S4B). Although these polymorphisms might not directly be located on primer binding sites, they might cause changes in the thermodynamics of template DNA, thus resulting in off-target or inefficient amplification. Another evidence of nucleotide polymorphisms that affect Dirus AS-PCR primer binding is demonstrated in Fig. S12A. Primer-BLAST results revealed that the binding site of D-AC primer on *An. dirus* ITS2 sequences misidentified as *An. baimaii* contains additional mismatch in addition to the 3′ single nucleotide mismatch.

Another essential factor influencing the accuracy of the Dirus AS-PCR primers is the handling during reaction setup. Taq polymerase is active at room temperature and can cause unintended amplification if the reaction is not kept on ice during the preparation. We found that the reaction setup had to be continuously conducted on ice to avoid nonspecific amplification. Hot-start enzymes can be a viable alternative to reduce unintended amplification, albeit with higher cost per reaction.

We found that the different primers of the previous set of Dirus AS-PCR had high GC content, which leads to high Tm. The recommended Ta of 51 °C in the protocol was much lower than the Tm of the original primers, thus increasing nonspecific binding. After optimization of the PCR conditions by increasing the temperature at the annealing step, coupled with reducing primer concentrations, PCR results were improved. Nevertheless, the specificity of the primers still hinders the use of this protocol to identify species within the *An. dirus* complex.

A thorough in silico analysis pinpointed problematic primers in the original Dirus AS-PCR. It allowed for the design of DiCSIP primers, which improved specificity, operational range, and sensitivity, enabling the accurate identification of all five member species of the GMS. Although requiring two separate reactions to differentiate all five species, it is essential to note that even with the original Dirus AS-PCR, a second SSP reaction is needed to distinguish between *An. dirus* and *An. scanloni*, as our investigation demonstrated, the Dirus AS-PCR had difficulty discriminating between these two species. The two-step PCR process has also been used to develop the multiplex PCR to identify five sibling species of the *An. barbirostris* complex [10].

Validation using laboratory and field samples demonstrated that DiCSIP offers correct identification even in different laboratories, using different Taq reagents and thermal cyclers, which show the reproducibility of the new primer set. The broad operational range and high specificity allow us to use the DNAzol direct reagent to process mosquito samples and use them directly in the PCR reaction without DNA extraction, thereby saving both time and cost in sample processing.

While it would have been ideal to include, in the development of DiCSIP, *An. aff. takasagoensis*, the sixth member of *An. dirus* complex found in the GMS, specifically in a restricted area of northern Vietnam [19], unfortunately its ITS2 sequence is not available in the NCBI database. This extends to *An. scanloni* and *An. nemophilous,* which also lack ITS2 sequence information, and only those in Walton et al. [9] are available. The need for molecular information on *An. dirus* complex members underscores a significant knowledge gap in the genetic information of these species. This lack of recent molecular and population genetic information can hinder efforts for effective and sustainable malaria vector control as it is impossible to evaluate whether the tools for species identification are still efficient.

## Conclusions

Taken together, this study addresses significant challenges in *An. dirus* complex identification by developing the DiCSIP assay improves the specificity and efficiency of species identification. The new primer set provides a valuable tool for accurate entomological surveys, supporting efficient vector control measures to reduce malaria transmission and prevent the reintroduction of the disease in the GMS region. The study emphasizes the importance of molecular information to develop tools for the reliable identification of sibling species of *Anopheles* complexes and improve malaria control strategies.

### Supplementary Information

Supplementary Material 1: Figure S1. The original dirus AS-PCR assay failed to identify members of the *An. dirus* complex in different laboratories. Figure S2. The dirus AS-PCR assay fails to consistently identify members of *An. dirus* complex even after condition optimization. Figure S3. In silico analysis reveals multiple potential dirus AS-PCR D-U universal forward primer binding sites. Figure S4. In silico analyses reveal the misidentification of *An. scanloni* as *An. dirus*. Figure S5. In silico analysis reveals multiple potential binding sites of the dirus AS-PCR species-specific D-AC, D-B, D-D, and D-F reverse primers. Figure S6. Multiple sequence alignment of ITS2 sequences of five species of the *An. dirus* complex and binding sites of primers used in DiCSIP. Figure S7. In silico analysis reveals only on-target potential binding sites of the DiCSIP-Uni-Fwd universal forward primer. Figure S8. In silico analysis reveals only on-target potential binding sites of the DiCSIP-Rev-AC Dirus/Scanloni-specific reverse primer. Figure S9. DiCSIP-Rev-C *An. scanloni*-specific reverse primers cannot be used for *An. scanloni* identification. Figure S10. In silico analysis reveals potential off-target binding sites of the DiCSIP-Fwd-C *An. scanloni*-specific forward primer. Figure S11. In silico analysis reveals potential off-target binding sites of the DiCSIP-Rev-F *An. nemophilous*-specific reverse primer. Figure S12. In silico analyses reveal misidentification of *An. dirus* as *An. baimaii*.

Supplementary Material 2: Table S1*.* Expected amplicon sizes of six *An. scanloni*-specific reverse primers when using with DiCSIP-Uni-Fwd primer.

## Data Availability

All data supporting the conclusions of this article are included within the article and supporting materials.

## Abbreviations

DiCSIP: Dirus complex species identification PCR
BIOTEC: National Center for Genetic Engineering and Biotechnology
GMS: Greater Mekong Subregion
AS-PCR: Dirus allele-specific PCR
PCR: Polymerase chain reaction
ITS2: Internal transcribed spacer 2
KU: Kasetsart University
AFRIMS: Armed Forces Research Institute of Medical Sciences
HLC: Human landing collection
gDNA: Genomic DNA
pDNA: Plasmid harboring ITS2 gene
DMSO: Dimethyl sulfoxide
Ta: Annealing temperature
TAE: Tris-acetate-EDTA
NCBI: National Center for Biotechnology Information
Tm: Melting temperatures
Fwd: Forward
Rev: Reverse
