# Supplementary material for: Dirus complex species identification PCR (DiCSIP) improves the identification of Anopheles dirus complex from the Greater Mekong Subregion
Source: Parasit Vectors. 2024 Jun 16;17:260. doi: 10.1186/s13071-024-06321-6 (PMC11181648; doi:10.1186/s13071-024-06321-6)
Supplement: Supplementary file 1 — Supplementary Material 1: Figure S1. The original dirus AS-PCR assay failed to identify members of the An. dirus complex in different laboratories. Figure S2. The dirus AS-PCR assay fails to consistently identify members of An. dirus complex even after condition optimization. Figure S3. In silico analysis reveals multiple potential dirus AS-PCR D-U universal forward primer binding sites. Figure S4. In silico analyses reveal the misidentification of An. scanloni as An. dirus. Figure S5. In silico analysis reveals multiple potential binding sites of the dirus AS-PCR species-specific D-AC, D-B, D-D, and D-F reverse primers. Figure S6. Multiple sequence alignment of ITS2 sequences of five species of the An. dirus complex and binding sites of primers used in DiCSIP. Figure S7. In silico analysis reveals only on-target potential binding sites of the DiCSIP-Uni-Fwd universal forward primer. Figure S8. In silico analysis reveals only on-target potential binding sites of the DiCSIP-Rev-AC Dirus/Scanloni-specific reverse primer. Figure S9. DiCSIP-Rev-C An. scanloni-specific reverse primers cannot be used for An. scanloni identification. Figure S10. In silico analysis reveals potential off-target binding sites of the DiCSIP-Fwd-C An. scanloni-specific forward primer. Figure S11. In silico analysis reveals potential off-target binding sites of the DiCSIP-Rev-F An. nemophilous-specific reverse primer. Figure S12. In silico analyses reveal misidentification of An. dirus as An. baimaii. [file 13071_2024_6321_MOESM1_ESM.docx]

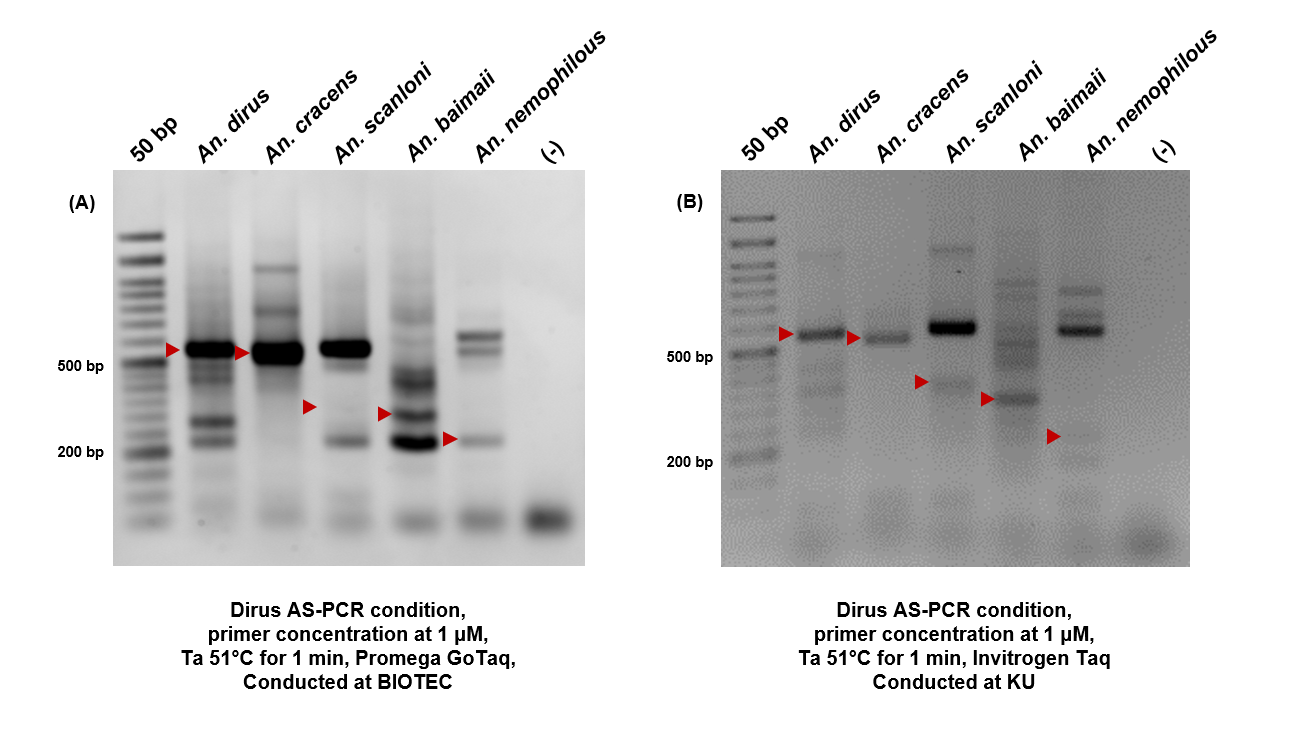


**Fig S1**. **The original Dirus AS-PCR assay fails to identify members of *An. dirus* complex in different laboratory.** Dirus AS-PCR was conducted following the exact conditions as described by Walton et al. (1999) (9) in two laboratories to identify *An. dirus*, *An. cracens, An. scanloni*, *An. baimaii*, and *An. nemophilous*. (A) AS-PCR conducted at BIOTEC using Promega GoTaq Flexi DNA Polymerase with BioRad C1000 Touch Thermal Cycler. (B) AS-PCR performed at KU using Invitrogen Taq DNA polymerase with Bioer LifePro Thermal Cycler. The red arrow mark indicates the expected sizes of PCR amplicons.


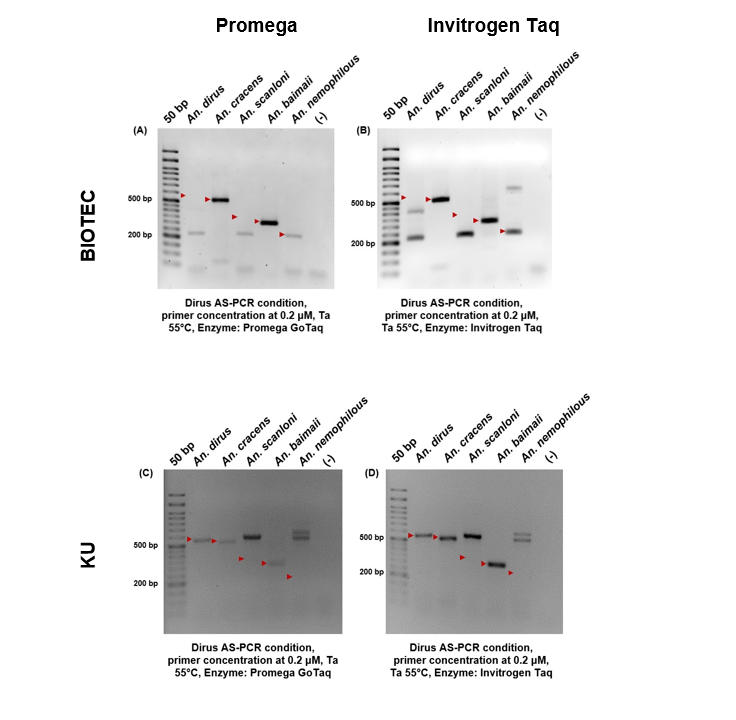


**Fig S2**. **The Dirus AS-PCR assay fails to consistently identify members of An. dirus complex even after condition optimization.** The Dirus AS- PCR was conducted using an optimized condition with different laboratories, Taq enzyme from different manufacturers and different thermal cyclers. The Dirus AS-PCR was performed at BIOTEC using Promega GoTaq Flexi DNA polymerase (A) and Invitrogen Taq DNA polymerase (B) with C1000 Touch Thermal Cycler, and at KU using Promega GoTaq Flexi DNA polymerase (C) and Invitrogen Taq DNA polymerase (D) with Bioer LifePro Thermal Cycler. The red arrow mark indicates the expected sizes of PCR amplicons.


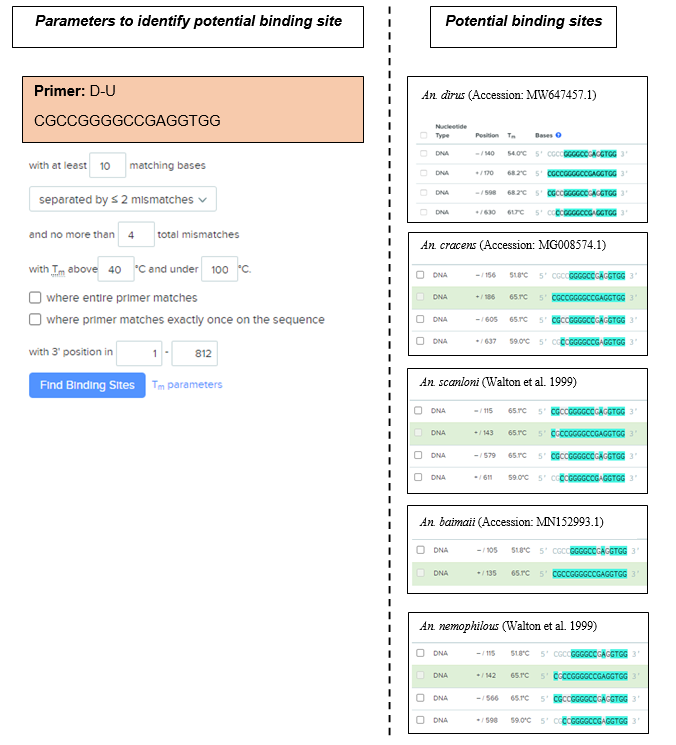


**Fig S3**. **In silico analysis reveals multiple potential binding sites of the Dirus AS-PCR D-U universal forward primer.** Potential binding sites of D-U were determined in An. dirus, An. cracens, An. scanloni, An. baimaii, and An. nemophilous ITS2 sequences using Benchling primer tool. The parameter used with this search includes 1) with at least 10 matching bases, 2) separated by ≤ 2 mismatches and no more than 4 total mismatches, 3) Tm above 40°C and under 100°C. The teal color indicates a matching bases between the primer and target sites.


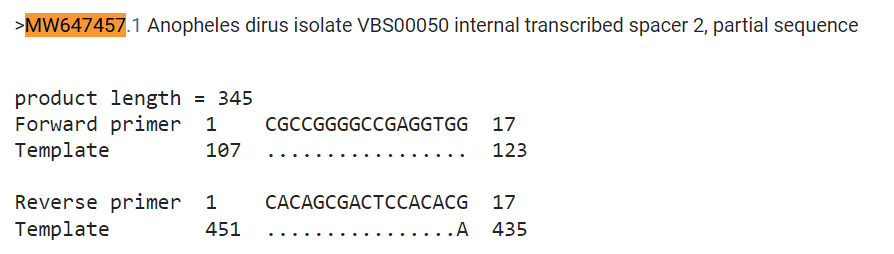

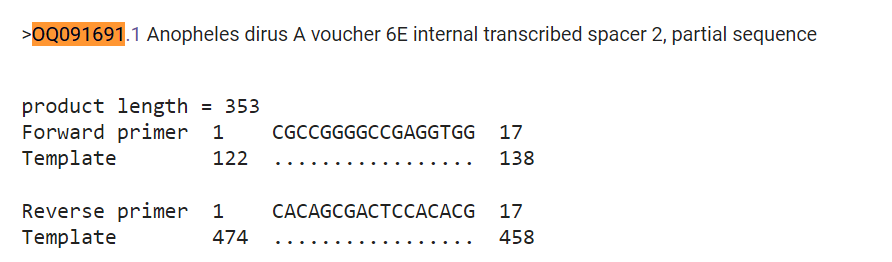


i

ii

iii

iv

v


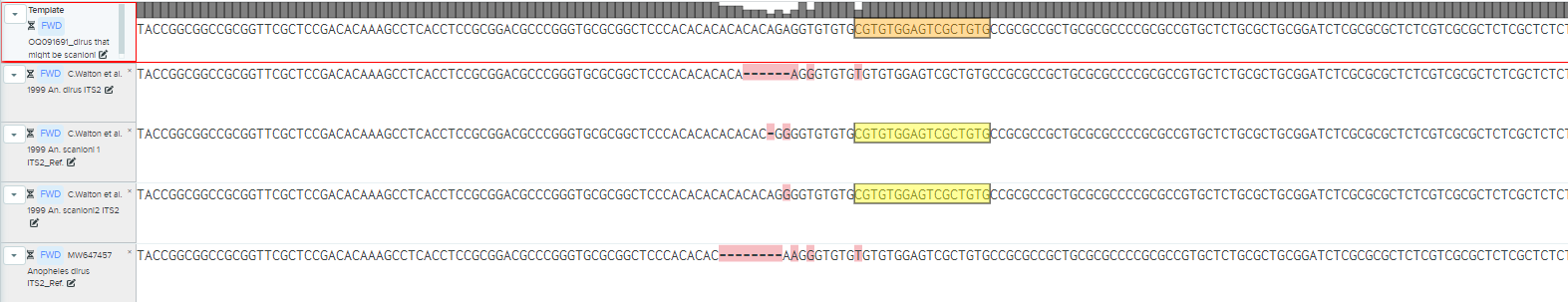


CGTGTGGAGTCGCTGTG

**D-AC**

**B**

Correctly identified *An. dirus* ITS2

*An. scanloni* ITS2 that was misidentified as *An. dirus*

**A**

**Fig S4**. **In silico analyses reveals misidentification of An. scanloni as An. dirus.** (A) Primer-BLAST results of the D-U and D-AC primers from Dirus AS-PCR reveals potential misidentification of An. scanloni as An. dirus. Complete match of D-AC reverse primer to ITS2 sequence from the database suggests misidentification of An. scanloni as An. dirus. Single nucleotide mismatch at 3’ end of D-AC reverse primer to ITS2 sequence from the database indicates that the sample is a correctly identified An. dirus. (B) Multiple Sequence Alignment of ITS2 sequence of An. dirus and An. scanloni demonstrated misidentification of An. scanloni as An. dirus. (i) A sequence of An. dirus obtained from the database which might be misidentified from An. scanloni. (ii) A sequence of An. dirus retrieved from Walton et al. (1999). (iii), (iv) A sequence of An. scanloni retrieved from Walton et al. (1999). (v) Sequences of An. dirus received from the database (MW647457). The yellow color indicates exact matches between the primer and target sites.


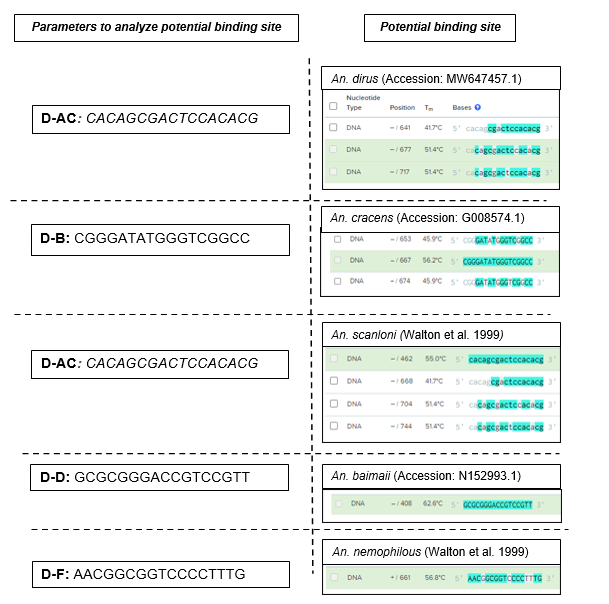


**Fig S5**. **In silico analysis reveals multiple potential binding sites of the Dirus AS-PCR species specific D-AC, D-B, D-D, and D-F reverse primers.** Potential binding sites of each primer were determined in ITS2 sequences of their respective target species using Benchling primer tool. The parameter used with this search includes 1) with at least 10 matching bases, 2) separated by ≤ 2 mismatches and no more than 4 total mismatches, 3) Tm above 40°C and under 100°C. The teal color indicates a matching bases between the primer and target sites


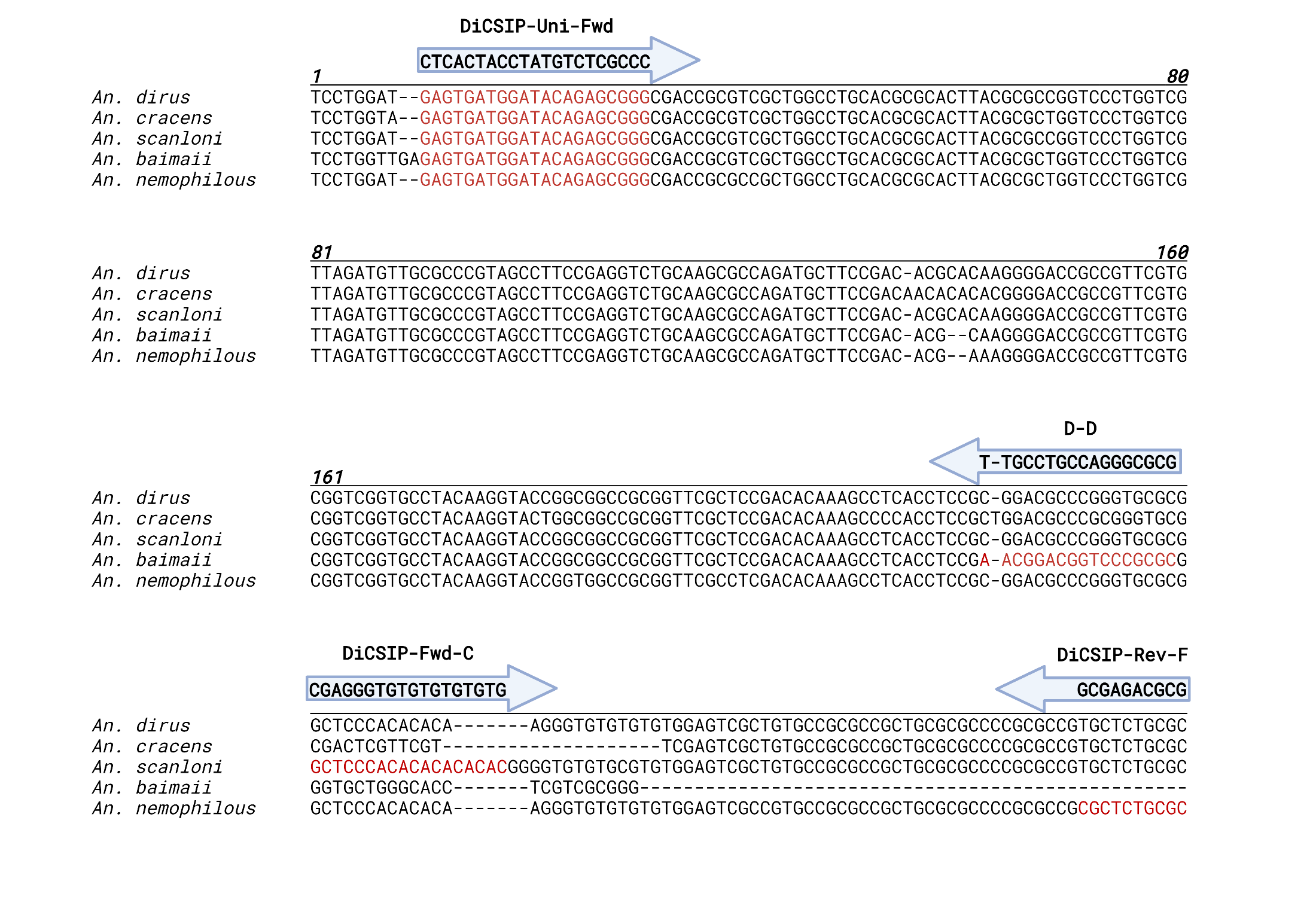


**
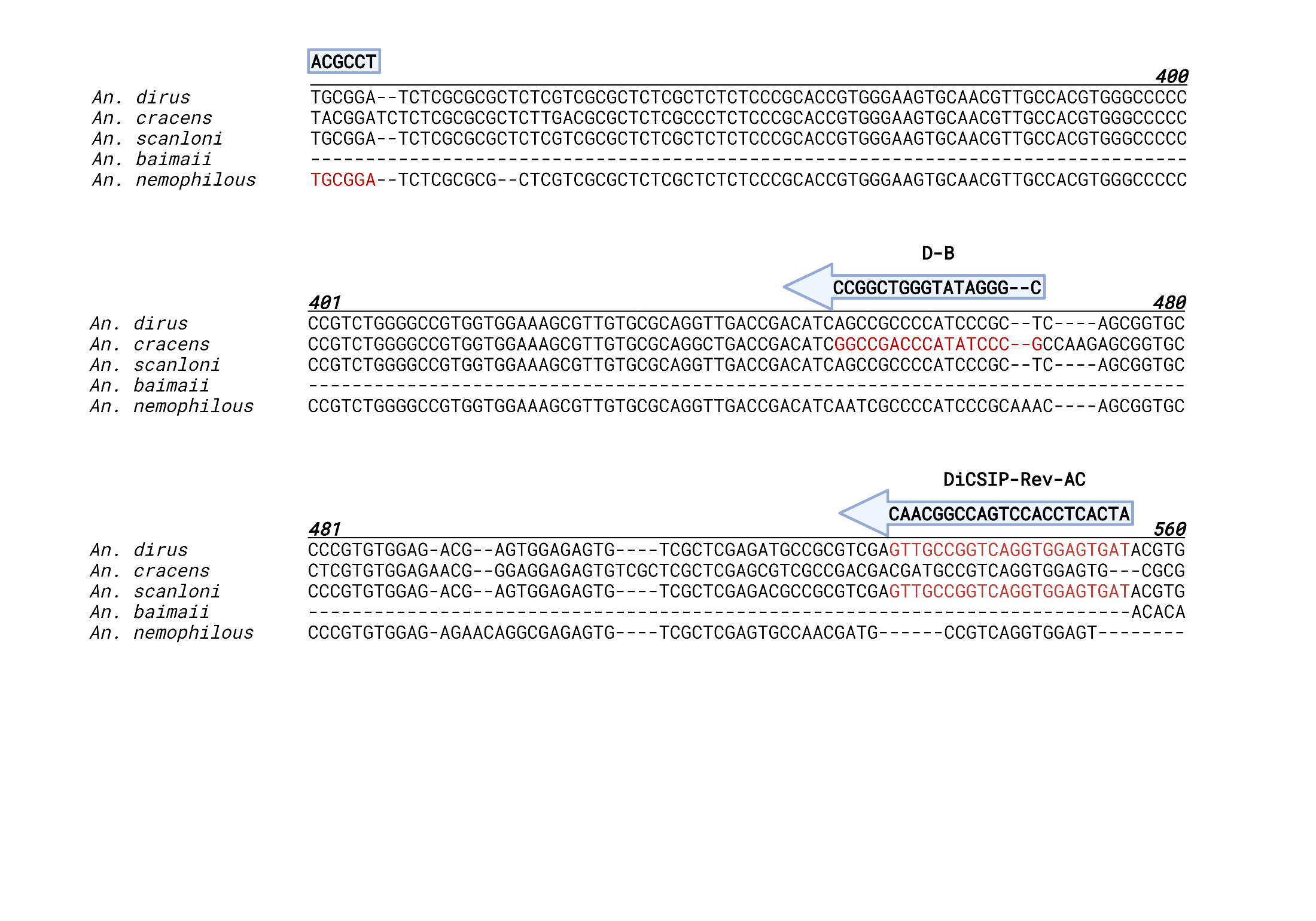
**

**Fig S6**. **Multiple sequence alignment of ITS2 sequences of five species of the An. dirus complex and binding sites of primers used in DiCSIP.** The sequences of An. dirus (Accession: MW647457.1), An. cracens (Accession: MG008574.1) and An. baimaii (Accession: MN152993.1) were retrieved from NCBI, while those of An. scanloni and An. nemophilous were obtained from a publish article (9). The correct binding sites of the primers were highlighted in red color letters.

**
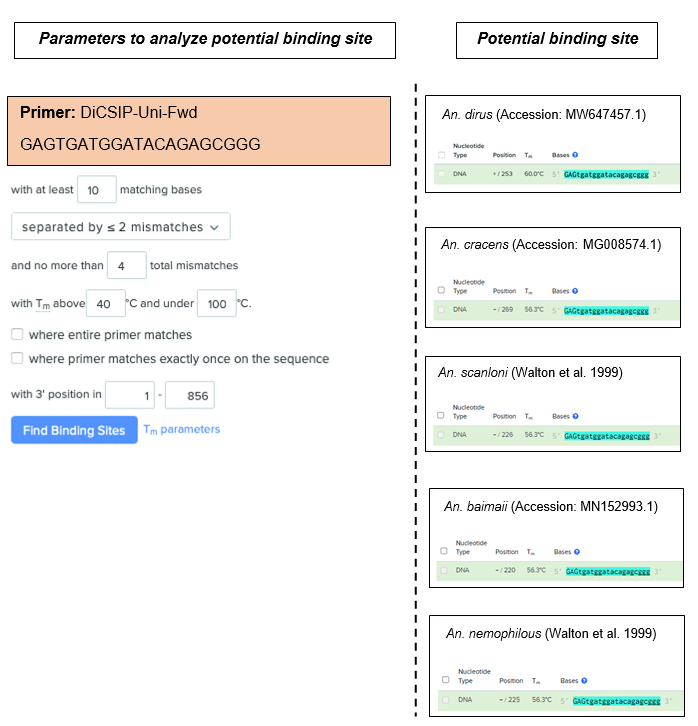
**

**Fig S7**. **In silico analysis reveals only on-target potential binding sites of the DiCSIP-Uni-Fwd universal forward primer.** Potential binding sites of DiCSIP-Uni-Fwd were determined in An. dirus, An. cracens, An. scanloni, An. baimaii, and An. nemophilous ITS2 sequences using Benchling primer tool. The parameter used with this search includes 1) with at least 10 matching bases, 2) separated by ≤ 2 mismatches and no more than 4 total mismatches, 3) Tm above 40°C and under 100°C. The teal color indicates a matching bases between the primer and target sites.


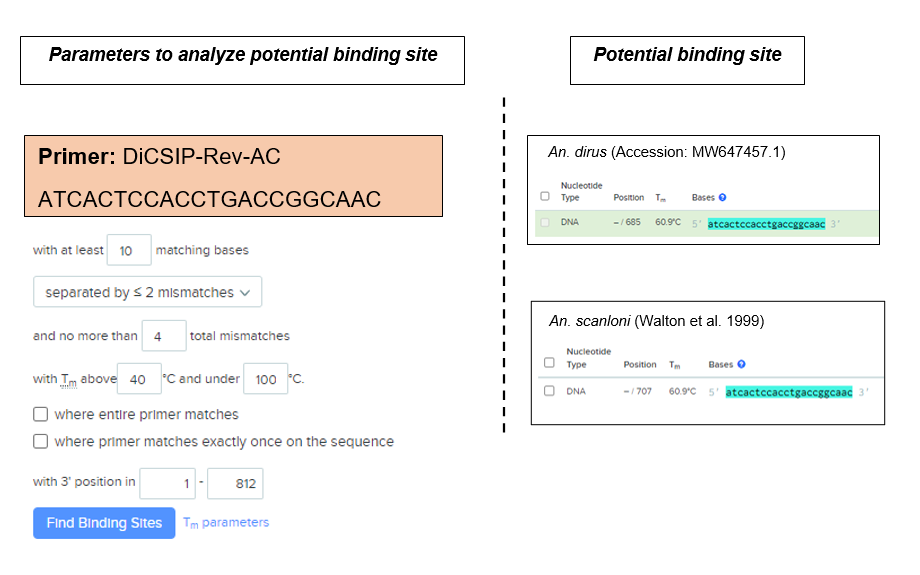


**Fig S8**. **In silico analysis reveals only on-target potential binding sites of the DiCSIP-Rev-AC Dirus/Scanloni specific reverse primer.** Potential binding sites of DiCSIP-Rev-AC were determined in An. dirus, and An. scanloni ITS2 sequences using Benchling primer tool. The parameter used with this search includes 1) with at least 10 matching bases, 2) separated by ≤ 2 mismatches and no more than 4 total mismatches, 3) Tm above 40°C and under 100°C. The teal color indicates a matching bases between the primer and target sites.

**
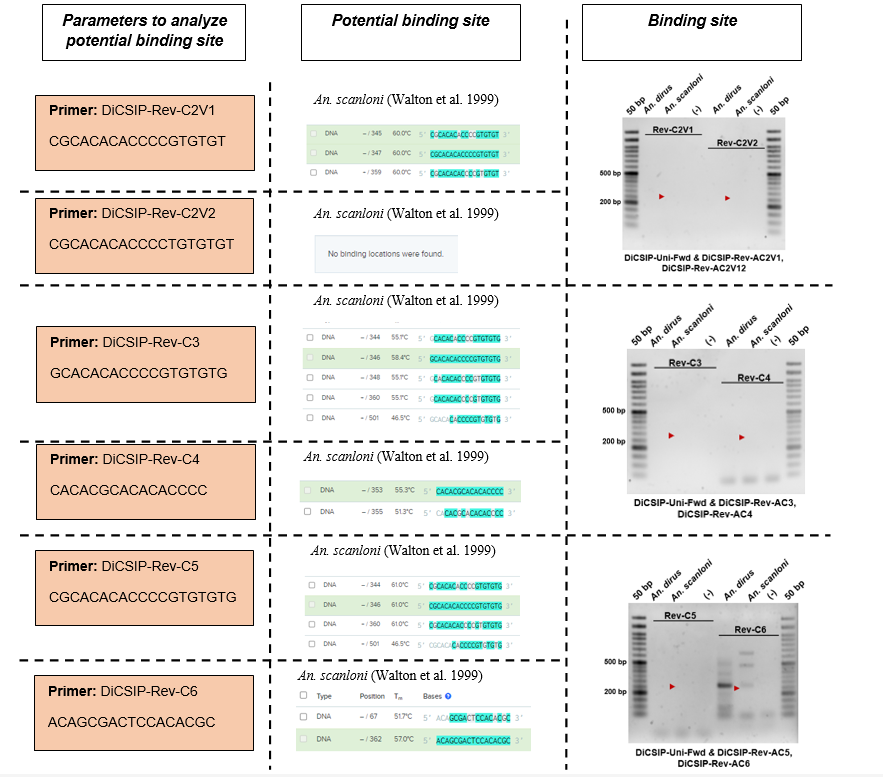
**

**Fig S9**. **DiCSIP-Rev-C An. scanloni specific reverse primers cannot be used for An. scanloni identification.** In silico analysis reveals off-target potential binding sites of the DiCSIP-Rev-C An. scanloni specific reverse primer. Potential binding sites of DiCSIP-Rev-C1-6 were determined in An scanloni ITS2 sequences using Benchling primer tool. The parameter used with this search includes 1) with at least 10 matching bases, 2) separated by ≤ 2 mismatches and no more than 4 total mismatches, 3) Tm above 40°C and under 100°C. The teal color indicates a matching bases between the primer and target sites. Six new reverse primers specific to An. scanloni were validated using a single-plex PCR to distinguish between An. dirus and An. scanloni. The red arrow mark indicates the expected sizes of PCR amplicons.

**
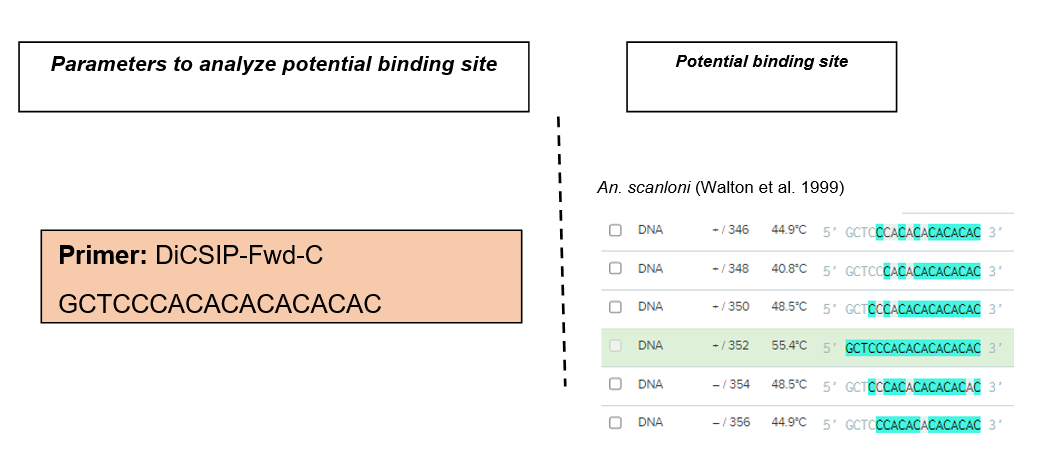
**

**Fig S10**. **In silico analysis reveals potential off-target binding sites of the DiCSIP-Fwd-C An. scanloni specific forward primer.** Potential binding sites of DiCSIP-Fwd-C were determined in An. scanloni ITS2 sequence using Benchling primer tool. The parameter used with this search includes 1) with at least 10 matching bases, 2) separated by ≤ 2 mismatches and no more than 4 total mismatches, 3) Tm above 40°C and under 100°C. The teal color indicates a matching bases between the primer and target sites.


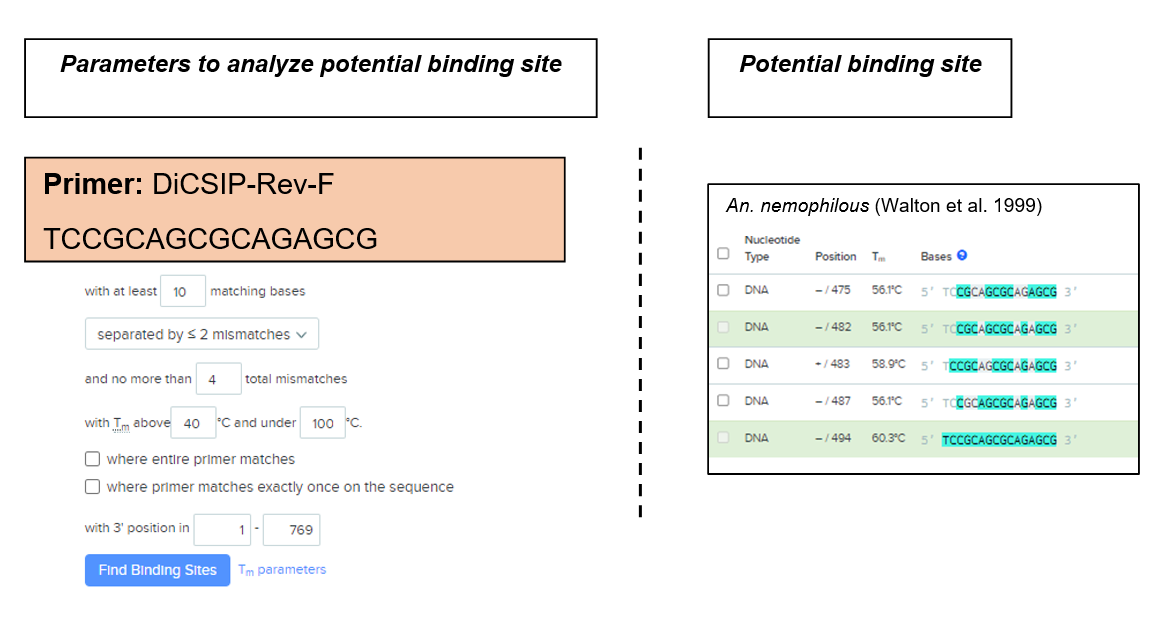


**Fig S11**. **In silico analysis reveals potential off-target binding sites of the DiCSIP-Rev-F An. nemophilous specific reverse primer.** Potential binding sites of DiCSIP-Rev-F were determined in An. nemophilous ITS2 sequence using Benchling primer tool. The parameter used with this search includes 1) with at least 10 matching bases, 2) separated by ≤ 2 mismatches and no more than 4 total mismatches, 3) Tm above 40°C and under 100°C. The teal color indicates a matching bases between the primer and target sites.


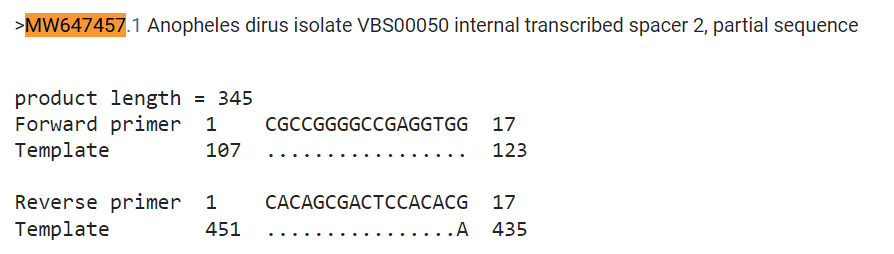

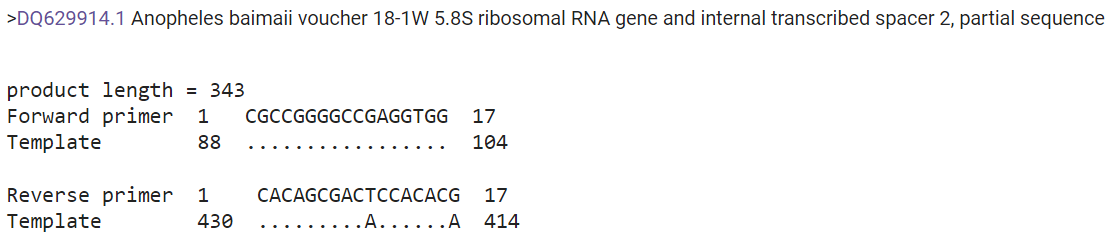


viii

AACGGACGGTCCCGCGC

**D-D**

i

ii

iii

iv

v


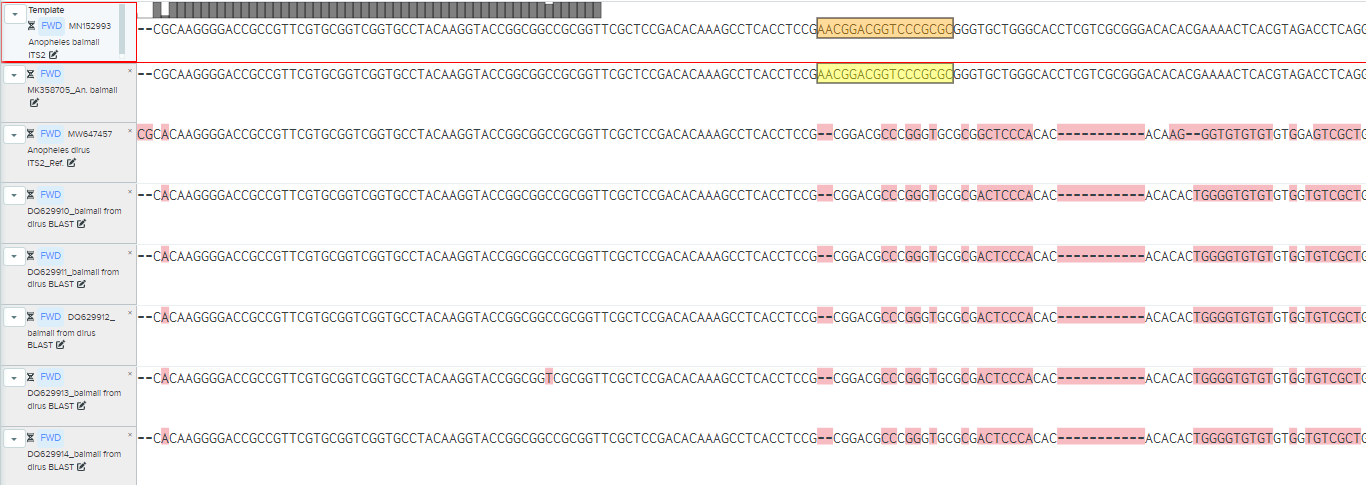


vi

vii

B

*An. dirus* ITS2 that was misidentified as *An. baimaii*

Correctly identified *An. dirus* ITS2

A

**Fig S12**. **In silico analyses reveal misidentification of An. dirus as An. baimaii.** (A) Primer-BLAST results of the D-U and D-AC primers from Dirus AS-PCR reveals potential misidentification of An. dirus as An. baimaii. (B) ITS2 sequence of An. dirus and An. baimaii was aligned in Benchling software with reverse D-D primer to observed potential misidentification of DNA sequence in NCBI database. (i) & (ii) Sequences of An. baimaii obtained from the database. (iii) A sequence of An. dirus retrieved from the database. (iv), (v), (vi), (vii) & (viii) Sequence of An. baimaii retrieved from the database which might be misidentification from An. dirus. The yellow color indicates a matching bases between the primer and target sites.
